# Agricultural Cyber-Physical Systems: Research Progress in Perception-Driven Multi-Robot Coordination and Logistics in Unstructured Environments

**DOI:** 10.3390/s26175514

**Published:** 2026-08-31

**Authors:** Jun Zhang, Tiantian Jing, Ziqi Tian, Honglei Zhang, Dong Lv, Zhong Tang

**Affiliations:** 1 School of Agricultural Engineering, Jiangsu University, Zhenjiang 212013, China; 2 School of Foreign Languages, Jiangsu University, Zhenjiang 212013, China

**Keywords:** agricultural cyber-physical systems (ACPS), multi-robot collaborative scheduling, digital twin and semantic mapping, human–robot collaboration, unstructured habitats, data-driven feedforward decision-making

## Abstract

Driven by the escalating global agricultural workforce shortage and the urgent need to meet the “Zero Hunger” mandate, the automation of harvest–transport workflows has emerged as a cornerstone of Agriculture 4.0. This paper highlights the latest research progress in multi-robot collaborative logistics scheduling across highly unstructured farming environments, underpinned by cutting-edge spatial perception and digital twin frameworks. Initially, we summarize the technological leap from conventional 2D geometric mapping to multi-modal semantic 3D reconstruction—fusing light detection and ranging (LiDAR), unmanned aerial vehicle (UAV) imagery, and spatial data—to enable high-fidelity forward-looking predictions. The discussion then transitions to algorithmic advancements, emphasizing the shift from traditional centralized operations research to decentralized, data-driven approaches such as Multi-Agent Reinforcement Learning (MARL). We also explore micro-kinematic predictive control mechanisms and the growing integration of ecological sustainability metrics into routing models. To demonstrate practical engineering progress, multi-agent implementations are analyzed across three typical spatial settings: high-throughput continuous relays in open fields, global navigation satellite system (GNSS)-denied discrete routing in dense orchards, and close-proximity human–robot collaboration (HRC) in smart greenhouses. Finally, we identify the remaining barriers to the large-scale commercialization of Agricultural Cyber-Physical Systems (ACPS), such as the “Sim-to-Real” gap restricted by edge-computing capacities, unclosed economic loops, and HRC ethical dilemmas, offering a forward-looking roadmap for next-generation resilient agricultural networks.

## 1. Introduction

Driven by the rapid growth of the global population and the ongoing progression of urbanization, modern agriculture is currently facing the dual, formidable challenge of significantly enhancing system productivity while simultaneously addressing severe labor shortages. In response to this global predicament, Sharma et al. have clearly pointed out that the Agriculture 4.0 paradigm—characterized by the deep integration of the Internet of Things (IoT), artificial intelligence, edge computing, and robotics—has emerged as a core driving force for achieving the global “Zero Hunger” objective [1]. Within this macro-evolutionary context, traditional agricultural production models are undergoing profound structural reshaping [2]. Fountas et al. provided a rigorous analysis of automation trends in field operations, emphasizing that due to the inherently labor-intensive and spatiotemporally repetitive nature of tasks such as harvesting and weeding, system-level operational entities are transitioning from individual automation toward distributed fleets of robots and heterogeneous multi-agent collaboration, which represents an inevitable trend for overcoming current agricultural productivity bottlenecks [3,4,5].

However, despite significant progress in underlying navigation control and microscopic perception for individual agricultural robots [6], systematic efficiency bottlenecks remain prominent in high-intensity farmland harvesting operations, primarily concentrated at the spatiotemporal interface between harvesting and transport. As revealed by the operations research and joint scheduling models developed by Wang et al. and Li et al., harvesters (or harvesting robots) and supporting transport vehicles exhibit highly significant heterogeneous characteristics in terms of operational speed, dynamic time windows, payload capacity, and spatial maneuverability, which frequently lead to capability mismatch under complex constraints [7,8].

In the absence of scientific dynamic scheduling mechanisms and optimized infield logistics planning, this heterogeneity directly leads to severe farmland logistics congestion and ecological degradation. On one hand, harvesters waiting for transport while at full capacity not only disrupt operational continuity but also result in the ineffective idle time of expensive machinery and a sharp increase in scheduling costs. On the other hand, the frequent and disorganized entry of transport vehicles into fields or their maneuvering during headland turning inevitably induces spatial path conflicts within the system and exacerbates the crushing of unharvested crops. Furthermore, studies on agricultural sustainability by Mier et al. (Soil2Cover) have confirmed that disordered machinery trajectories cause significant soil compaction [9], whereas the introduction of coverage path planning that accounts for spatiotemporal coordination can reduce headland soil compaction by as much as 30% [10].

Therefore, in highly dynamic and unstructured farmland environments, constructing a multi-objective optimization model that balances low latency, low energy consumption, and minimal ecological damage to achieve efficient spatiotemporal matching of heterogeneous harvest–transport equipment groups has become the most critical pain point constraining the scale-up and sustainability of modern agricultural production.

To fundamentally resolve the aforementioned harvest–transport logistics bottleneck, it is imperative to dismantle the system boundaries of isolated agricultural machinery operation. As clearly pointed out by Benos et al., modern agricultural equipment systems are undergoing a comprehensive evolution toward Agricultural Cyber-Physical Systems (ACPS), aiming to achieve highly efficient coupling and closed-loop control between physical agricultural machinery entities and computational communication networks [11]. Under the ACPS architecture, the harvest–transport cluster has moved beyond the simple physical superposition of multiple machines. Lujak et al. precisely reformulated such agricultural vehicle scheduling problems into decentralized, highly dynamic Agriculture Fleet Vehicle Routing Problems (AF-VRP). In this multi-agent network, each agricultural mobile robot (AMR) serves as both an execution endpoint and an independent agent equipped with distributed perception and adaptive negotiation capabilities [12]. Furthermore, research by Purcell and Wang et al. indicates that with the aid of Digital Twin (DT) technology, the system has begun to transition from “digital shadows” with unidirectional data flows to “complete digital twins” possessing bidirectional closed-loop feedback mechanisms [13,14]. Through high-fidelity modeling, DT technology accurately maps the spatiotemporal dynamics of physical farmland into a virtual computational space, thereby providing the entire multi-agent fleet with a “nerve center” for advanced prediction, what-if simulation, and global optimal coordination [15].

A survey of top-tier research in the field of agricultural robotics in recent years reveals a distinct evolutionary trajectory in theory and technology: a comprehensive leap from “micro-level automation” at single nodes to “multi-machine collaborative scheduling and control” at the system level. In their latest systematic review, Wei et al. pointed out that while early research was highly concentrated on environmental perception and bottom-level trajectory tracking of single-machine platforms, recent technological breakthroughs have deeply focused on spatiotemporal networking and conflict-free collaboration of heterogeneous agricultural machinery (such as harvesters and supporting grain-transport vehicles, or heterogeneous UGV and UAV formations) in complex field and orchard scenarios [16].

Accompanying this paradigm shift, the logical core of underlying multi-machine collaborative algorithms has undergone a qualitative reconstruction. Early fleet scheduling was often based on rigorous static environmental prior assumptions and centralized rule-based operations. However, real farmland environments are replete with dynamic obstacles, complex terrain, and non-linear disturbances. As confirmed by the research of Ye et al. and Wang et al., traditional deterministic planning algorithms (such as standard DWA or Pure Pursuit) often reveal defects such as rigid weighting, poor environmental adaptability, and a lack of robustness when dealing with such unstructured, high-interference conditions [17,18]. Consequently, the academic focus is accelerating toward perception-driven artificial intelligence paradigms. Rather than relying on static prior maps, modern Deep Reinforcement Learning (DRL) models continuously ingest high-frequency sensor streams—such as LiDAR-derived local occupancy grids and visual semantic features—to adaptively output continuous navigation weights in completely unknown environments [18,19]. In the dimension of multi-agent interaction, distributed sensor networks and edge-computed perception data are now seamlessly integrated with learning algorithms, enabling fleets to achieve collaborative collision avoidance based strictly on real-time sensory feedback rather than rigid central commands [20]. This algorithmic transformation from passive rule-based response to active feedforward prediction and reinforcement learning represents the current highest academic frontier in multi-machine collaborative scheduling and autonomous decision-making in unstructured agricultural environments [21,22].

Although research on harvest–transport fleet collaboration is growing exponentially, existing literature is often limited to confined to fragmented discussions within isolated technical domains. There remains a severe lack of a systematic review capable of bridging the entire end-to-end chain, from “bottom-level multi-modal collaborative perception” to “middle-level dynamic collaborative scheduling algorithms” and “top-level multi-scenario logistics practice.” To fill this important academic gap, this paper focuses deeply on multi-machine collaborative logistics scheduling in complex, unstructured farmland environments, aiming to construct a global technical map encompassing perception, planning, control, and scheduling.

The subsequent sections of this paper are organized as follows: Section 2 deeply explores the “data foundation” of cluster scheduling systems, systematically analyzing the multi-modal collaborative perception fusion mechanism in complex unstructured environments and the Digital Twin (DT) modeling technology evolving from digital shadows to bidirectional closed-loop control. Section 3, serving as the core theoretical section, systematically reviews the algorithmic paradigm shift from traditional Operations Research Optimization to data-driven Feedforward Prediction, and provides an in-depth analysis of micro-level path conflict resolution and high-precision obstacle avoidance strategies based on vehicle kinematic constraints. Section 4 situates the theoretical framework within the frontiers of agricultural application, deeply analyzing logistics scheduling paradigms in three typical complex agricultural scenarios: continuous flow in open fields, discrete flow in orchards, and high-density spatial collaboration in greenhouses. Section 5 moves beyond a purely technical perspective to provide a forward-looking exploration of the comprehensive feasibility and challenges of agricultural multi-robot systems from multi-dimensional macro-scales, including economic benefits, social acceptance, and legal and ethical norms, followed by a summary and outlook.

## 2. Literature Search Strategy and Selection Criteria

To systematically review the latest research progress in multi-robot collaborative logistics scheduling across highly unstructured farming environments, a rigorous literature search and screening strategy was established.

### 2.1. Search Strategy and Data Sources

A comprehensive literature retrieval was conducted across several major scientific databases, including Web of Science, Scopus, IEEE Xplore, and ScienceDirect. The search primarily focused on recently published articles to accurately capture the paradigm shift from traditional individual automation toward distributed fleets of robots and heterogeneous multi-agent collaboration. The core search string incorporated specific keywords directly aligned with the thematic focus of this review, including “agricultural cyber-physical systems (ACPS)”, “multi-robot collaborative scheduling”, “digital twin and semantic mapping”, “human-robot collaboration”, “unstructured habitats”, and “data-driven feedforward decision-making”. These primary terms were systematically combined with related concepts such as edge computing, multimodal perception, and operations research using appropriate Boolean operators to ensure a broad yet highly relevant retrieval scope.

### 2.2. Inclusion and Exclusion Criteria

To ensure the high academic quality and thematic alignment of the selected studies, specific inclusion and exclusion criteria were rigorously applied in a continuous evaluation process. Included studies were required to explicitly address multi-robot collaborative logistics scheduling and control underpinned by cutting-edge spatial perception and digital twin frameworks. Furthermore, selected articles had to propose algorithmic advancements, such as the shift from traditional centralized operations research to decentralized, data-driven approaches like Multi-Agent Reinforcement Learning (MARL). Practical engineering implementations were also required, meaning the studies had to evaluate systems in typical agricultural spatial settings, including high-throughput continuous relays in open fields, global navigation satellite system (GNSS)-denied discrete routing in dense orchards, or close-proximity human–robot collaboration (HRC) in smart greenhouses. Conversely, studies were strictly excluded if they purely focused on operations in structured, highly controllable industrial warehousing environments that lack the intense unstructured characteristics of real-world farmland. Research limited solely to micro-level automation or the bottom-level trajectory tracking of single-machine platforms without addressing multi-machine collaborative scheduling at the system level was also excluded. Non-peer-reviewed technical reports, preprints, and non-English publications were similarly omitted to maintain the rigorous academic standard of this review.

### 2.3. Literature Screening Process

The screening process followed a systematic multi-stage approach to distill the most relevant literature. Initially, duplicate records obtained from the various databases were identified and removed. In the preliminary screening phase, the titles and abstracts of the retrieved articles were independently reviewed by the authors to eliminate studies that clearly fell within the exclusion criteria. Following this, full-text assessments were conducted on the remaining articles to verify their depth and relevance to the core topics of agricultural cyber-physical systems. Only studies that successfully passed this rigorous final evaluation were integrated into the review. This systematic process ensured the selection of high-quality literature capable of bridging the entire end-to-end chain, from bottom-level multi-modal collaborative perception to middle-level dynamic collaborative scheduling algorithms and top-level multi-scenario logistics practice.

## 3. Data Foundation: Multi-Modal Perception and Digital Twin Modeling in Unstructured Environments

The accuracy and robustness of macro-level decision-making (e.g., cross-regional task allocation and dynamic path planning) in any efficient cluster logistics scheduling system are inevitably dependent on the high fidelity and ultra-low latency of underlying input data. Unlike structured, highly controllable industrial warehousing environments—which feature flat, rigid surfaces, seamless global Wi-Fi coverage, and pre-installed fiducial markers [23]—real-world farmland environments exhibit intense unstructured and highly dynamic spatiotemporal succession. Specifically, high-density crop canopy occlusion, muddy and rugged unpaved terrain, drastic light intensity fluctuations, and phenotypic characteristics that evolve continuously with the crop’s physiological stages [24] constitute severe physical interference. This significant situational barrier dictates that agricultural multi-robot systems cannot rely on a single sensing modality; instead, they must construct deep multi-sensor fusion architectures (such as Visual-Inertial-LiDAR, or VIL-Fusion) [25] to ensure that fleets can achieve precise pose estimation and seamless local environmental perception via Collaborative SLAM technology, even in GNSS-degraded or denied environments such as poly-tunnels or high-density orchards. More crucially, to bridge the semantic gap between massive, unstructured underlying perception data streams and upper-level operations research decision engines, the system must transform multi-dimensional, fragmented perceptual features in real-time into Digital Twin (DT) models that scheduling algorithms can directly compute and invoke [26].

Based on the aforementioned system-level architectural requirements, this section focuses deeply on the data foundation of logistics scheduling systems, aiming to analyze how to construct a digital interaction bridge that spans physical farmland and virtual computational space. The content of this section unfolds along two core dimensions: “distributed collaborative localization and edge intelligence” and “3D semantic extraction and twin modeling of farmland.”

First, we focus on the multi-agent collaborative perception mechanism in complex farmland environments with weak signals. This section systematically discusses how edge computing architectures are deployed at agricultural machinery nodes (empirical studies show that by relying on low-power edge devices such as the NVIDIA Jetson, the latency of high-precision visual inference in open fields can be drastically compressed to the magnitude of 0.0549 s). Simultaneously, it deeply dissects frontier human–robot collaborative localization frameworks such as “Navigate-and-Seek,” analyzing how they achieve a 4.7-fold improvement in topological localization accuracy for personnel and dynamic targets in complex occluded environments compared to traditional methods by fusing Topological Particle Filters (TPF) with sub-optimal active perception strategies (Next-Best-Sense, NBS).

Second, we systematically dissect the efficient extraction of physical semantics in farmland environments and the construction of the digital twin foundation. We explore how to utilize improved algorithms such as FAST-LIO2 and LeGO-LOAM, combined with traversability analysis, to eliminate high-frequency noise from voluminous 3D point clouds (e.g., based on Patchwork++ and Z-score statistical filtering) and precisely extract traversable geometric boundaries with clear kinematic constraint significance, such as support columns and canopy borders. Finally, we detail how digital twin systems break through the limitations of the “digital shadow” (which only offers unidirectional data mapping) to construct a complete digital twin possessing bidirectional closed-loop control and what-if simulation capabilities [27,28], thereby providing a high-fidelity and robust data cornerstone for the complex global logistics coordination and data-driven feedforward predictive scheduling discussed in subsequent sections. Table 1 summarizes representative perception and localization technologies tailored for unstructured agricultural environments, along with their core technical mechanisms, applicable scenarios and validated performance metrics.

### 3.1. Distributed Collaborative Localization and Intelligent Perception

**Figure 1:**
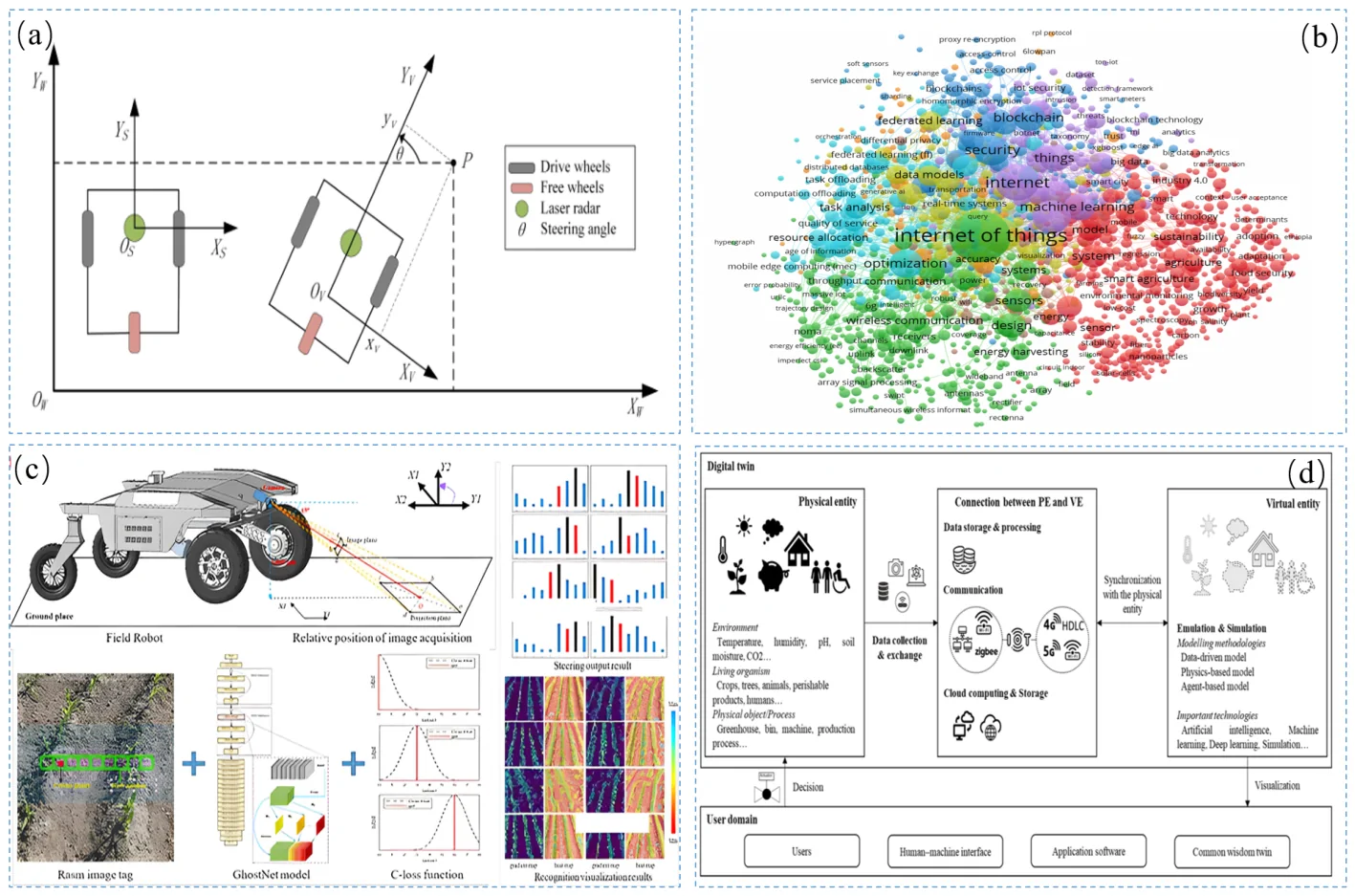
**The digital foundation of the agricultural harvesting-transport logistics scheduling system: From multi-modal perception to digital twin architecture.** (**a**) The relationship between robot, LiDAR, and global coordinate system [36]. (**b**) Vision and LiDAR fusion pipeline for crop row semantic extraction and navigation control [37]. (**c**) Flowchart of vision-based line detection in a corn field [38]. (**d**) Architectural framework of agricultural digital twins [13].

In the complex engineering architecture of multi-agent fleet collaboration, achieving high-frequency and high-precision estimation of single-machine global absolute poses, dynamic perception of multi-machine relative spatial configurations, and real-time 3D reconstruction of environmental geometric topologies and obstacle boundaries is an insurmountable prerequisite for the scheduling hub to execute macro path planning and dynamic task allocation [31]. However, the real physical environment of farmland exhibits intense unstructured characteristics, posing a severe challenge to traditional surveying-grade localization methods. Especially under high-density canopy occlusion and severe multipath effects induced by complex, non-paved undulating terrain, signals from the Global Navigation Satellite System (GNSS) are highly prone to high-frequency attenuation or even prolonged loss (i.e., GNSS-denied/degraded environments). Addressing this thorny bottleneck in navigation blind spots, passive responses relying solely on single-platform sensors have suffered from severe performance degradation. Therefore, constructing a multi-modal deep fusion architecture based on Visual-Inertial-LiDAR (VIL-Fusion), introducing IoT wide-area networking mechanisms, and promoting the edge-computing offloading of underlying computational power constitute the core technological foundation for breaking through the perception barriers of unstructured farmland [32,33,34,35]. As illustrated in Figure 1, the digital foundation of the agricultural harvesting-transport logistics scheduling system relies heavily on transitioning from isolated to multi-modal perception. Figure 1a,b demonstrate the relationship between the robot and the global coordinate system, alongside the vision and LiDAR fusion pipeline essential for extracting crop row semantics. Furthermore, Figure 1c,d detail the vision-based line detection flowchart in a corn field and the comprehensive architectural framework of agricultural digital twins, which collectively construct the essential data bridge spanning physical farmland and virtual computational spaces.

At the physical construction level of the macro-perception network, the underlying data collection paradigm of the system is undergoing a substantive transition from isolated proprioception to distributed connected perception. The inherent limitation of a single-machine perspective is that its perceptual boundaries are easily truncated by line-of-sight (LoS) blockages, whereas heterogeneous multi-machine networking (such as UGV and UAV collaboration) effectively breaks this physical constraint. Addressing this evolutionary theoretical architecture, Soussi et al. systematically reviewed multi-modal intelligent data acquisition technologies in precision agriculture, profoundly revealing that the deep coupling of Internet of Things (IoT) communication protocols, artificial intelligence inference algorithms, and underlying smart sensor networks [39] serves as the core driving force for modern precision agriculture to optimize system resource allocation and achieve wide-area, high-fidelity interconnection of physical agricultural variables. Adapting to this trend of total-factor networking, the recent empirical study by Mansoor et al. further enriches the digital connotation of perception networks. Their work demonstrates that the seamless integration of intelligent sensing terminals and IoT frameworks not only constructs a highly robust Decision Support System (DSS) for Agriculture 4.0 [40], but also enables an order-of-magnitude leap in the cluster network’s capability for real-time monitoring and characterization of underlying physical states, such as spatiotemporal microclimate succession and soil moisture dynamics [41,42]. Consequently, this provides a real-time and consistent global state benchmark for the upper-level harvest–transport logistics scheduling engine [37].

However, under traditional computing architectures heavily reliant on cloud-centric processing, the long-distance transmission of massive multi-modal perception data—especially 3D point clouds and high-resolution video streams—inevitably incurs significant communication latency and channel jitter. This poses a fatal systemic response bottleneck for heterogeneous harvest–transport clusters that are highly dependent on high-frequency state interactions and millisecond-level dynamic closed loops. To thoroughly dismantle this data transmission barrier, research by Akhtar et al. deeply demonstrates the decisive role of edge computing in wide-area data processing for modern smart agriculture [43]. This study elucidates that by offloading core AI inference and data preprocessing capabilities directly to sensor nodes or agricultural machinery edge-side devices near the data source, the system can physically evade congestion in remote backbone data links [44] (empirical studies show that relying on low-power edge-side devices, the inference latency of complex vision models in field environments can be drastically compressed to an extremely low magnitude of 0.0549 s), thereby greatly satisfying the real-time constraints of high-frequency multi-machine collaborative localization. Furthermore, targeting high-dimensional visual perception streams that easily consume communication bandwidth, Talib et al. systematically reviewed video data management models within agricultural edge computing environments [45]. Their in-depth analysis explicitly points out that pre-deploying high-computing-power video cascading processing nodes at the agricultural machinery terminal side not only significantly reduces the bandwidth load on the backbone network, but also lays a vital distributed computing architectural foundation for the cluster to achieve efficient distributed collaborative SLAM, decentralized dynamic obstacle avoidance, and ultra-low-latency sharing of key semantic feature streams within local area networks [46].

Shifting the focus downward to the microscopic single-machine localization and execution level, the observational limitations of a single sensor are completely exposed when facing high-interference physical environments in farmland. Therefore, constructing front-end odometry algorithms based on strict spatiotemporal synchronization and tight coupling of multi-source heterogeneous data has become the mainstream paradigm for breaking through the robustness bottlenecks of single-machine localization. To address the challenges of long-term localization drift and pose degradation easily induced in highly dynamic, unstructured environments, Yao et al. developed a multi-modal perception framework deeply fusing vision, inertial measurement units (IMUs), and LiDAR (VIL-Fusion) [47]. This study innovatively introduced advanced semantic segmentation and point cloud filtering mechanisms into the front-end odometry, which not only effectively eliminated leaves and weeds that easily sway with the wind and generate high-frequency dynamic noise (i.e., non-rigid interference), but also precisely extracted thick, geometrically stable tree trunks as high-quality global static landmarks [48,49]. Through the deep decoupling of dynamic environmental interference and continuous local optimization, this system substantially suppressed the accumulated relative pose error during long-term operations on unpaved, rugged terrain, providing a high-fidelity state estimation benchmark for long-term autonomous single-machine navigation in complex farmland scenarios [50,51].

When dealing with drastic illumination variations and high-frequency intrusions of dynamic obstacles in unstructured farmland, the perception blind spots of a single sensor are often severely amplified [52,53]. To address this pain point, Han et al. proposed a heterogeneous perception framework based on the deep synergy of 3D LiDAR and camera vision [36]. Through high-precision extrinsic calibration and strict spatiotemporal synchronization of underlying data streams, this study achieved feature-level alignment between dense 3D point clouds and texture-rich 2D RGB images [54]. This mechanism not only achieves high-fidelity boundary reconstruction and topological extraction of dynamic and static obstacles in orchards within a 3D metric space, but also thoroughly compensates for the algorithmic foundation for the pixel feature loss and matching failures easily induced by vision-only sensors when facing intense backlight, overexposure, or mottled woodland light and shadows. While this multi-modal complementary mechanism significantly enhances anti-interference capabilities under extreme lighting conditions [55], a critical horizontal comparison reveals a major methodological flaw in current fusion architectures: the immense computational overhead. High-fidelity alignment of dense 3D point clouds with high-resolution 2D images demands extreme processing power, creating a severe technological bottleneck when deployed on low-cost, resource-constrained agricultural edge devices. Consequently, the current research gap lies in developing lightweight, asymmetric fusion algorithms that can balance perceptual accuracy with the strict energy constraints of field robots.

Furthermore, beyond multi-modal deep fusion at the algorithmic dimension, the scalability and cross-platform plug-and-play compatibility of the underlying hardware architecture are also core considerations determining whether high-level perception systems can achieve large-scale deployment across various heterogeneous agricultural equipment. Based on this engineering and industrial-grade requirement, Shamshiri et al. designed and validated a distributed modular perception architecture based on the Controller Area Network (CAN-bus) protocol [56]. This system efficiently integrates and synchronizes multi-dimensional observation signals from multi-channel infrared sensor arrays and Time-of-Flight (ToF) laser rangefinders at the physical data acquisition layer, while innovatively deploying a low-latency exponential filtering algorithm directly at the edge of independent Electronic Control Units (ECUs). This mechanism, which relies on underlying hardware computing power for edge data preprocessing, successfully isolates sensor observation outliers and high-frequency white noise caused by dense, cluttered foliage occlusions from the physical source. Empirical results demonstrate that even when the mobile robot frequently traverses dense berry bushes with severely constrained structural space, the system can still output highly stable and reliable local obstacle avoidance vector commands and relative localization references, greatly enhancing the real-time responsiveness and engineering applicability of the underlying actuators.

Breaking through the limitations of traditional agricultural machinery constrained to single-dimensional planar observation, the multi-modal perception space of modern “harvest–transport” fleets is undergoing a leapfrog extension toward 3D Heterogeneous Collaborative Perception. Addressing the static Field of View (FoV) blind spots experienced by single ground mobile robots (UGVs) constrained by low-level perspectives and dense crop occlusion, Roldán et al. creatively deployed a UGV-UAV Collaborative System in complex greenhouse environments [57,58,59]. This architecture, relying on high-bandwidth, low-latency edge communication links, achieves deep synergy between large-scale, unobstructed macro-optimization by aerial drones (UAVs) and high-precision micro-feature capture by ground unmanned vehicles. This complementary mechanism not only successfully constructs a high-fidelity 3D spatiotemporal topological map containing farmland environmental variables such as temperature, humidity, light intensity, and CO2 concentration, but also significantly improves the spatial resolution and data coverage dimensions of the perception network. For upper-level fleet logistics scheduling, this “three-dimensional digital foundation” provides indispensable global prior constraints for resolving multi-machine collision-free trajectory planning in unstructured environments, dynamic allocation of cross-regional transport resources, and spatiotemporal feature matching in high-dimensional space.

On the other hand, with the deep embedding of the Human–Robot Collaboration (HRC) paradigm in field and compact greenhouse systems, the precise perception and tracking of highly dynamic human targets within dense clusters are no longer merely auxiliary indicators for optimizing global scheduling efficiency, but an absolute prerequisite for constructing physical interaction safety barriers. Facing the technical bottleneck in poly-tunnels where traditional passive perception easily loses targets due to severe occlusion by high-density canopies, Polvara et al. proposed a breakthrough “Navigate-and-Seek” active perception framework [60]. In its underlying pose estimation, this study abandoned continuous metric-space mapping—which is highly prone to failure—and innovatively introduced a Topological Particle Filter (TPF), which was deeply coupled with the Next-Best-Sense (NBS) algorithm based on multi-criteria decision-making. Under this mechanism, the robot can calculate the information gain of its field of view in real-time, proactively reconstruct its own kinematic configuration to acquire an optimal observation perspective, and perform bidirectional global and local state fusion with low-cost, ubiquitous GNSS nodes at the IoT foundation layer. Empirical data show that this heterogeneous perception paradigm—transitioning from “passive reception” to “active exploration”—improved the topological tracking accuracy of harvesting workers in complex occluded environments by 4.7 times compared to traditional methods [61]. For complex “harvest–transport” scheduling systems, this extremely robust dynamic spatiotemporal benchmark directly empowers the machinery fleet to perform precise dynamic dispatching of turnover boxes and seamless recovery of full or empty boxes to specific workers within intricate physical road networks, truly realizing an efficient closed-loop for human–machine collaborative logistics.

### 3.2. Farmland Semantic Extraction and Digital Twin Modeling

In the complex spatiotemporal collaborative operations of multi-agent fleets, relying solely on low-level geometric features captured by physical sensors—such as disordered point cloud coordinates or discrete 2D image grayscale values—exhibits significant perceptual limitations and information redundancy. Given that upper-level distributed scheduling algorithms struggle to directly parse such raw, bottom-level data lacking topological associations and physical constraints, the system must perform deep feature decoupling and knowledge mapping. This process transforms unstructured geometric data streams into high-dimensional environmental representations endowed with explicit physical properties and terrain traversability metrics. Table 2 classifies and compares mainstream path planning and obstacle avoidance paradigms for agricultural mobile robots, covering their algorithmic principles, core advantages and applicable constraints.

Based on this, this section systematically expounds the paradigm evolutionary path of farmland spatial environment modeling: we primarily discuss how environmental representation transitions from traditional, semantically impoverished 2D Occupancy Grid Maps to high-fidelity 3D Semantic Topological Maps that deeply integrate non-paved ground slip resistance, time-varying dynamic features, and low-lying random obstacles. Ultimately, this evolves into a full-dimensional Digital Twin (DT) foundation that comprehensively integrates multi-source, ubiquitous heterogeneous data streams and crop growth physiological models. The construction of this bidirectional, closed-loop “digital foundation” not only enables a highly robust mapping from physical farmland to virtual computational space but also constitutes an indispensable bottom-level data prerequisite for the cluster logistics scheduling hub to execute feedforward predictive coordination and global optimization of wide-area transport capacity resources [53].

At the level of lightweight extraction of local farmland environmental semantics and efficient deployment at the edge-terminal, the deep integration of machine vision technology and deep learning models (such as lightweight convolutional neural networks) has demonstrated significant potential for engineering applications [65,66,67,68,69,70]. Regarding open-field crop scenarios with relatively clear structural features, Bai et al. and Shi et al. provided systematic and comprehensive reviews of vision-based agricultural vehicle autonomous navigation and guidance technologies [38,71]. These landmark studies consistently demonstrate that, under harsh operating conditions characterized by complex illumination fluctuations and high-density weed interference, utilizing advanced computer vision architectures to precisely extract crop row centerlines [72,73] (crop row detection & centerline extraction) is not only the physical cornerstone for constructing local spatial semantic topologies in open fields [74,75,76,77], but also the underlying control core ensuring that various heterogeneous agricultural machines execute precise row-following operations and high-frequency trajectory dynamic correction [78,79,80].

However, when the perceptual boundary extends to high-density orchard environments with cluttered backgrounds and severe canopy occlusion, traditional multi-stage visual navigation algorithms—which are highly dependent on manual feature extraction and complex curve fitting—frequently encounter robustness degradation and generalization bottlenecks due to susceptibility to dynamic light spots and weed noise. To thoroughly resolve this perception pain point, Liu et al. proposed a highly forward-looking Single-Stage Navigation Path Extraction Network (NPENet) [81]. This architecture creatively abandons the redundant pixel-level mask segmentation and time-consuming morphological post-processing found in traditional visual paradigms, directly reconstructing the centerlines and vanishing points of unstructured inter-row paths as end-to-end prediction targets for deep neural networks. Empirical studies show that under severe edge-terminal computational resource constraints, NPENet can achieve a path centerline detection accuracy of up to 92.14% with an ultra-low single-frame inference latency of 10.1 milliseconds. This breakthrough provides ultra-low latency and high-fidelity local visual semantic priors for heterogeneous multi-machine fleets executing high-frequency, high-dynamic micro-trajectory scheduling within extremely narrow orchard rows.

Furthermore, beyond the construction of macro-path topologies, a refined characterization of microscopic environmental semantics is indispensable, given the physical characteristics of non-paved off-road terrain and the resulting intense non-linear disturbances to the underlying vehicle dynamics of agricultural machinery. To this end, Yao et al. developed a deep semantic segmentation framework named PPM-Unet [48]. Relying on the large-scale ASO3600 unstructured farmland geomorphology dataset constructed for this study, the model achieved, for the first time, high-precision pixel-level semantic perception of typical low-lying obstacles in open fields, such as random puddles, muddy slip zones, and uneven mounds. This achievement fundamentally breaks through the representational limitations of traditional 3D topological maps, which were previously confined to geometric obstacle avoidance. By deeply integrating microscopic geomorphological features into map data, it endows the upper-level logistics scheduling hub with the capacity for “Traversability Cost Representation”—a capability with profound physical significance. Based on these multi-dimensional underlying semantics, the fleet scheduling system can transcend blind geometric optimization, proactively predict, and dynamically avoid high-resistance danger zones prone to tire slip, bogging, and other losses of kinematic feasibility, thereby significantly enhancing the anti-disturbance capability and high-intensity operational continuity of the entire harvest–transport logistics chain [82,83].

Accompanied by the continuous refinement and feature decoupling of multi-dimensional environmental semantic representation, the data foundation of underlying logistics scheduling systems is undergoing a substantive transition from one-way, passive data monitoring (i.e., “Digital Shadow”) to a “Complete Digital Twin (DT)” architecture capable of high-fidelity bidirectional closed-loop control and advanced state prediction [84]. Targeting this frontier evolutionary trajectory, Purcell et al. provided a profound analysis of the paradigm-reconstructing potential of digital twin technology in complex agricultural scenarios [14]. Their research explicitly indicates that by relying on ubiquitous IoT infrastructure and high-fidelity digital mapping of physical farmland state elements, the fleet scheduling hub is thoroughly freed from delayed physical responses, enabling multi-threaded “what-if simulation” and strategy exercises within the virtual computational space. This feedforward trial-and-error mechanism based on virtual space fundamentally dismantles the high cost-of-failure barriers of physical systems, making it possible to achieve globally optimized dynamic allocation of multi-machine logistics transport capacity and agricultural resources (e.g., water, fertilizer, chemicals) under complex spatiotemporal constraints [85,86].

Regarding the specific construction mechanism of physical attribute mapping in the twin foundation, the latest comprehensive review by Zhang et al. (A Comprehensive Review of Digital Twins Technology in Agriculture) emphasizes the fundamental core position of constructing full-lifecycle 3D crop population evolution models [53]. This review confirms that by deeply assimilating multi-source heterogeneous datasets—including meteorological fluctuations, spatiotemporal dynamics of soil microclimates, and crop phenotypic development—the spatiotemporal prediction generalization capability of digital twin models over macro-time scales increases exponentially. However, their analysis rigorously points out critical engineering barriers for large-scale deployment [87,88,89]. More importantly, a critical methodological flaw in current Digital Twin literature is the severe lack of quantitative validation regarding the discrepancy between simulated dynamics and physical reality. While “what-if” simulations offer theoretically optimal schedules, current studies rarely quantify the micro-level physics divergence—such as exact metric deviations caused by non-linear tire-soil slip, transient sensor noise distributions, or unmodeled canopy aerodynamics. The absence of strict quantitative error boundaries (e.g., specific Root Mean Square Errors (RMSE) between simulated trajectory predictions and actual field execution) renders many current DT models as ideal mathematical abstractions rather than robust engineering tools.

**Figure 2:**
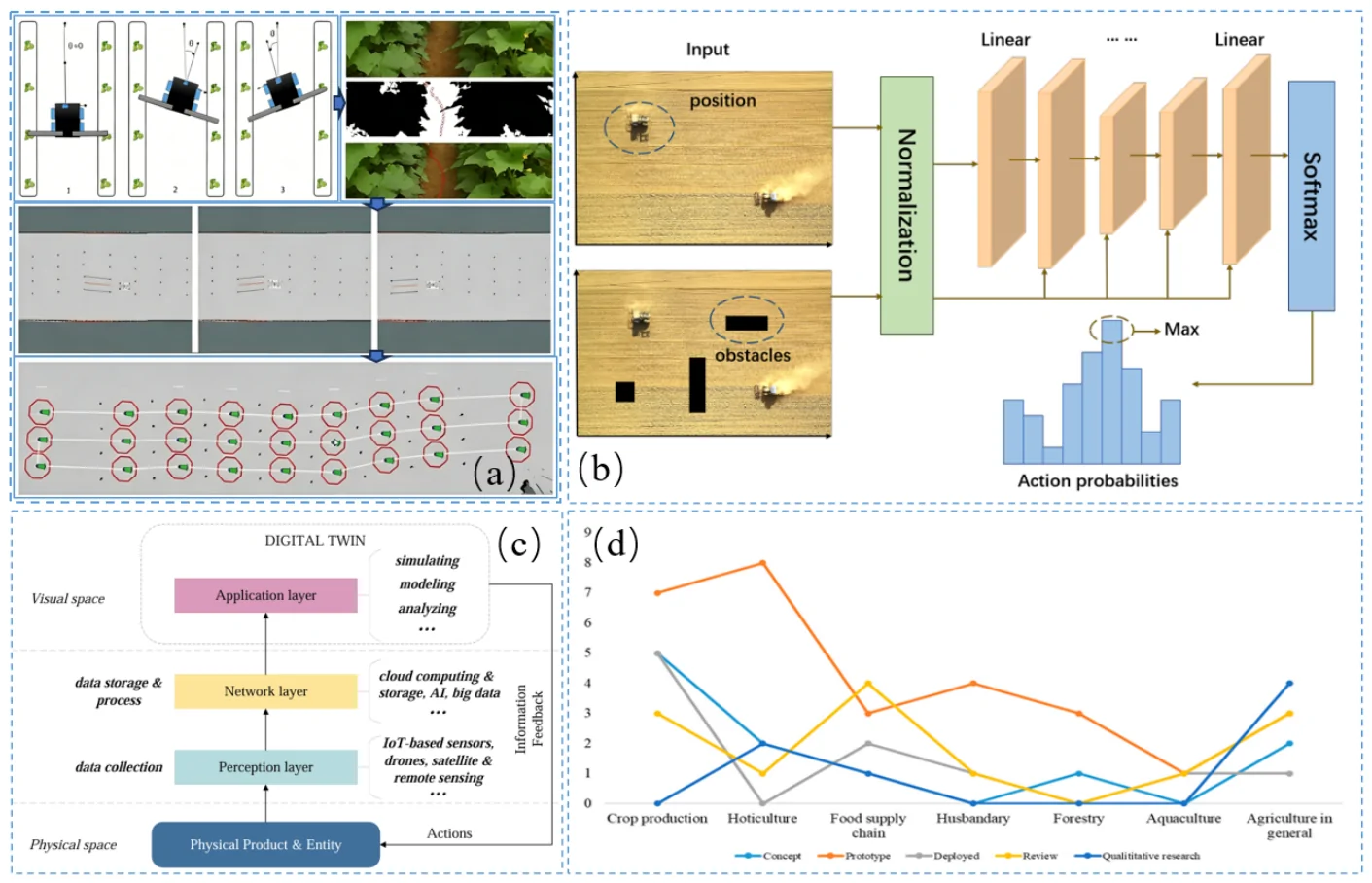
**The comprehensive technical pipeline for multi-dimensional semantic extraction and bi-directional closed-loop Digital Twin (DT) modeling in agriculture.** (**a**) Applications of navigation-based crop row detection in greenhouses [38]. (**b**) The residual-like network used in reinforcement learning [19]. (**c**) The architecture of Digital Twins and functions of each layer [53]. (**d**) Types of publications in different agricultural domains [13].

A more groundbreaking academic frontier lies in the substantive deep coupling between the static digital twin foundation and top-level macro-operations scheduling algorithms, which has nurtured a new generation of logistics paradigms driven by “physical perception, twin evolution, and intelligent scheduling.” Zhang et al. (Multiple agricultural machinery cooperative operations) proposed a multi-machine collaborative task allocation and path planning framework with highly dynamic environmental adaptability [90]. This model thoroughly breaks through the limitations of traditional scheduling based solely on geometric distance, innovatively reconstructing yield production data based on remote sensing inversion as core environmental constraint variables, and deeply embedding them into the underlying logic of improved Ant Colony Optimization (ACO) and Conflict-Based Search (CBS) for multi-machine planning. This coordination strategy, which fully integrates “yield prior semantic maps” into the twin foundation, endows the system with an extremely powerful feedforward deduction capability—namely, the ability to proactively and precisely predict the absolute physical locations and optimal relay time windows where heavy combine harvesters reach full-bin thresholds (unloading points) within specific spatial topologies of the farmland. This achievement represents a global first in realizing the advanced, high-precision matching of dynamic load constraints of heterogeneous fleets with the physical semantics of farmland space, marking the formal advance of unstructured farmland logistics scheduling toward a phase of highly intelligent holographic cognitive coordination. To further visualize this comprehensive technical pipeline, Figure 2 depicts the multi-dimensional semantic extraction and bi-directional closed-loop Digital Twin (DT) modeling in agriculture. Specifically, Figure 2a,b exhibit the applications of navigation-based crop row detection and the residual-like network utilized in reinforcement learning. Meanwhile, the multi-layered DT architecture and the distribution of publications across different agricultural domains are outlined in Figure 2c,d, emphasizing the profound evolution towards highly intelligent holographic cognitive coordination.

## 4. Core Algorithms: Global Scheduling and Local Collaboration for Harvest–Transport Fleets

Relying on the mapping support from the aforementioned high-fidelity environmental perception and multi-dimensional digital twin foundation, the “computational brain” of multi-machine cluster collaborative scheduling formally assumes its pivotal role in global control. From the deep modeling perspective of systems engineering and operations research, the spatiotemporal collaborative operation of agricultural “harvest–transport” clusters in complex farmland environments is essentially a typical NP-hard combinatorial optimization problem characterized by high nonlinearity, strong coupling, and complex constraints. The critical challenge in breaking through this technical bottleneck lies in achieving deep-coupled decision-making across global task allocation, sequential optimization of collaborative processes, and 3D spatial path topology selection, all while operating under rigorous and high-concurrency physical constraints—including high-urgency crop physiological harvest time windows, asymmetric payload capacity limits of heterogeneous agricultural machinery, and highly unstructured, dynamically evolving farmland topologies.

Based on the aforementioned system evolution requirements, this section serves as the core algorithmic deduction hub of this paper, aiming to systematically deconstruct current frontier paradigms in the field of agricultural multi-machine cluster scheduling. The detailed discussion follows a logical thread from macro-task coordination to micro-physical execution, systematically deconstructed into three progressive technical branches:

First, the Macro-task Allocation and Multi-objective Optimization Layer: We deeply review the optimization mechanisms of traditional operations research modeling and modern meta-heuristic algorithms (such as improved Ant Colony Optimization, NSGA-III, and hybrid Marine Predators Algorithms (IMPA)) in solving high-dimensional multi-objective scheduling problems. We specifically discuss how scheduling strategies are transitioning from simple efficiency-driven models to agricultural ecological paradigms that deeply integrate soil sustainability constraints (such as the Soil2Cover ecological compaction minimization criteria).

Second, the Data-driven Collaborative Decision Layer: We systematically dissect the data-driven framework centered on reinforcement learning, particularly the disruptive potential of Multi-Agent Reinforcement Learning (MARL) and Soft Actor-Critic (SAC) algorithms in handling sudden disturbances and achieving decentralized feedforward predictive coordination. This paradigm effectively compensates for the lack of robustness in traditional deterministic algorithms when dealing with unstructured terrain interference, significantly enhancing the autonomous negotiation and decision-making adaptability of fleets in unpredictable environments.

Finally, the Micro-kinematic Constraint and Collaborative Conflict Resolution Layer: We ground the macro-scheduling blueprint in micro-physical execution, meticulously deconstructing the kinematic mapping strategies following the issuance of high-level instructions [91]. We focus on analyzing local trajectory refinement under non-holonomic kinematic constraints for articulated agricultural machinery (such as tractor-trailer collaborative systems), dynamic deadlock-free conflict resolution based on Conflict-Based Search (CBS) algorithms, and robust path-tracking technology based on Nonlinear Model Predictive Control (NMPC) [92]. This cross-level mapping mechanism ensures that top-level logistics collaborative decisions can ultimately be transformed into high-stability, high-precision real-time operational execution instructions for terminal agricultural machinery. Table 3 summarizes the state-of-the-art cooperative scheduling and motion control approaches for agricultural machinery fleets, in terms of algorithmic framework, optimization objectives and typical application scenarios.

From the perspective of architectural evolution, the development of harvest–transport fleet scheduling algorithms has witnessed a distinct paradigm shift from centralized operations to decentralized control, and both frameworks retain their respective positions in current research and engineering practice. Centralized scheduling architectures, represented by classical operations research models and meta-heuristic algorithms, rely on a unified computing hub to collect global state information and generate globally optimal solutions for all agents in a top-down manner. This paradigm still maintains irreplaceable application value in scenarios with high environmental controllability, stable task flows, and limited fleet scales: it delivers mathematically rigorous global optimal solutions and features low deployment complexity for small and medium-sized farms with fixed operation boundaries.

However, as operation scales expand, heterogeneous agent types proliferate, and dynamic disturbances in unstructured farmland intensify, the inherent limitations of centralized architectures have become increasingly prominent: exponential growth in computational complexity with fleet size, vulnerability to single-point failures of the control center, and insufficient real-time responsiveness to abrupt local disturbances. Driven by these bottlenecks, decentralized scheduling paradigms represented by Multi-Agent Reinforcement Learning (MARL) and distributed model predictive control have developed rapidly. Under such architectures, each agricultural machinery node acts as an independent intelligent agent that perceives its local environment, negotiates with neighboring agents, and makes autonomous decisions, which significantly improves system scalability, fault tolerance, and dynamic response speed.

Notably, there is no absolute substitution relationship between the two paradigms in current research and industrial practice. In real engineering deployments, a hybrid architecture of “global centralized coarse scheduling + local decentralized fine-tuning” is widely adopted: the centralized operations research engine completes macro-level global task allocation and path topology planning, while decentralized intelligent algorithms handle micro-level dynamic obstacle avoidance and real-time conflict resolution. This complementary logic explains why centralized methods still occupy an important position in the current literature.

### 4.1. Operations Research Optimization and Heuristic Task Allocation

In the field of Global Operations Research Optimization, achieving efficient decoupling and precise allocation of operational tasks under high-concurrency dynamic constraints—thereby ensuring strict load balancing of heterogeneous fleet resources and minimizing non-productive costs (such as unnecessary deadheading, deadlock-induced waiting, and energy redundancy) [96]—remains a core scientific proposition in the design of cluster scheduling systems. Early academic exploration was largely confined to static rule mapping and centralized analytical computation; however, as the physical boundaries of operations evolve toward large-scale, multi-field collaborative scenarios [97], these traditional deterministic algorithms are prone to the “Curse of Dimensionality” when facing the exponentially exploding combinatorial search space. To break through this computational bottleneck, multi-objective evolutionary computation, equipped with powerful global parallel search capabilities, and meta-heuristic algorithms incorporating deep fusion mechanisms have replaced traditional paradigms to become the mainstream architecture for solving such high-dimensional, strongly coupled, and non-linear combinatorial optimization problems [98,99,100].

Within the rigorous modeling framework of spatiotemporal operations research, Ali et al. pioneered foundational exploration into the logistics bottlenecks of field harvesting operations [101]. By constructing rigorous multi-constraint mathematical models and vehicle routing scheduling algorithms based on graph theory, they achieved global collaborative route planning for heavy-duty combine harvesters and grain-transport vehicles [102]. Building on this, modern scheduling architectures have shifted to rely entirely on real-time data acquired from widespread agricultural sensor networks. For instance, by processing multimodal data streams—such as harvester bin-level sensor readings, real-time GNSS trajectories, and machine vision-based crop density estimations—Li et al. established a multi-objective optimization model that dynamically updates time windows and payload limits [8]. Their solution leverages these continuous sensory inputs to drive heuristic algorithms, demonstrating that robust scheduling in unstructured fields is fundamentally dependent on the low-latency acquisition and fusion of field sensor data. Simulation and empirical results demonstrate that this hybrid paradigm not only effectively reduces system configuration redundancy but also exhibits extremely high computational robustness and operational efficiency under complex time-varying constraints, providing a powerful algorithmic cornerstone for scientific decision-making in harvest logistics.

Furthermore, to bridge the gap between theoretical models and the complex engineering requirements of unstructured farmland, the underlying logic of cluster scheduling algorithms is accelerating its evolution toward paradigms of “deep multi-dimensional constraint fusion” and “high-frequency dynamic response.” Targeting the problem of spatial conflict during the collaborative harvesting and grain-transfer process, Wang et al. constructed a refined collaborative model integrating high-dimensional kinematic boundaries and proposed a Hybrid Genetic-Heuristic Iterative (HGHI) algorithmic structure. The core engineering innovation of this paradigm lies in the introduction of a “Dynamic unloading point relocation strategy” based on farmland spatial topological boundaries [7]. By forcing the full-load interaction between heterogeneous agricultural machines to take place in the headland zone, this strategy mathematically and topologically avoids the severe soil compaction and crushing of unharvested crops caused by transport vehicles frequently penetrating the physical interior of the farmland. This coordination architecture, which explicitly incorporates “soil compaction cost” into the scheduling cost function, marks the formal entry of unstructured agricultural logistics scheduling into a phase of end-to-end coordination that balances operational efficiency, computational robustness, and the ecological sustainability of cultivated land [103].

Along the logical thread of complex constraint decoupling, Liu et al. explored the dynamic collaborative operational boundaries between tracked corn harvesters and transport fleets, constructing a dual-objective operations research optimization model aimed at simultaneously minimizing global scheduling costs and makespan, while adapting it with an enhanced hybrid multi-objective evolutionary algorithm (MOEA/D-LSA) [104]. This study rigorously quantified multi-dimensional and stringent constraints, including the physical payload limits of heterogeneous agricultural machinery, transient energy consumption models, micro-level safety collision-avoidance distances, and rigid agronomic harvest time windows. Relying on its built-in high-frequency state monitoring and local path re-planning mechanisms, this strategy endows the fleet with extremely high robustness in task reallocation and trajectory correction when facing extreme dynamic disturbances, such as unknown farmland obstacles or sudden equipment failure. To thoroughly break the latency of traditional scheduling algorithms in spatiotemporal deduction, the research by Zhang et al. injected an advanced feedforward coordination capability into the algorithm [90]. This team proposed a multi-machine collaborative task allocation architecture with strong external dynamic adaptability; beyond integrating and reconstructing an improved ant colony optimization (ACO) algorithm at the foundation, its most prominent theoretical contribution lies in the innovative extraction of farmland spatial yield production data—based on multi-spectral satellite remote sensing inversion—as core spatiotemporal constraint variables, which were then deeply embedded into the system’s evolutionary algorithm architecture. This mechanism is a global first in overcoming the challenge of proactively and precisely matching fleet logistics dynamic loads with the macro-level spatial yield semantics of farmland, marking a fundamental leap in scheduling paradigms from “passive response” to “active optimization.”

Furthermore, within the generalized evaluation dimension of systems engineering, the scheduling objectives of modern Agricultural Cyber-Physical Systems (ACPS) are no longer limited to pure spatiotemporal execution efficiency; “Ecological Sustainability” is becoming a new gold standard for measuring the engineering value of algorithms. The pioneering work of Mier et al. directly addresses this pain point, proposing an eco-friendly macro-path planning and task scheduling paradigm named Soil2Cover [10]. This research is the first to directly mathematize the complex underlying soil compaction dynamics (relying on the high-fidelity SoilFlex model) and deeply couple them into the cost objective function of global routing algorithms as a core penalty term [105]. By strictly limiting the frequency of heavy machinery traversing specific vulnerable soil regions in the time domain, this topological optimization strategy not only achieved a 4% to 6% reduction in total system non-productive travel distance but also surprisingly slashed headland soil structural compaction degradation by up to 30%, thereby establishing a solid theoretical benchmark for constructing a green, environmentally minimal-resistance, and sustainable fleet scheduling system.

**Figure 3:**
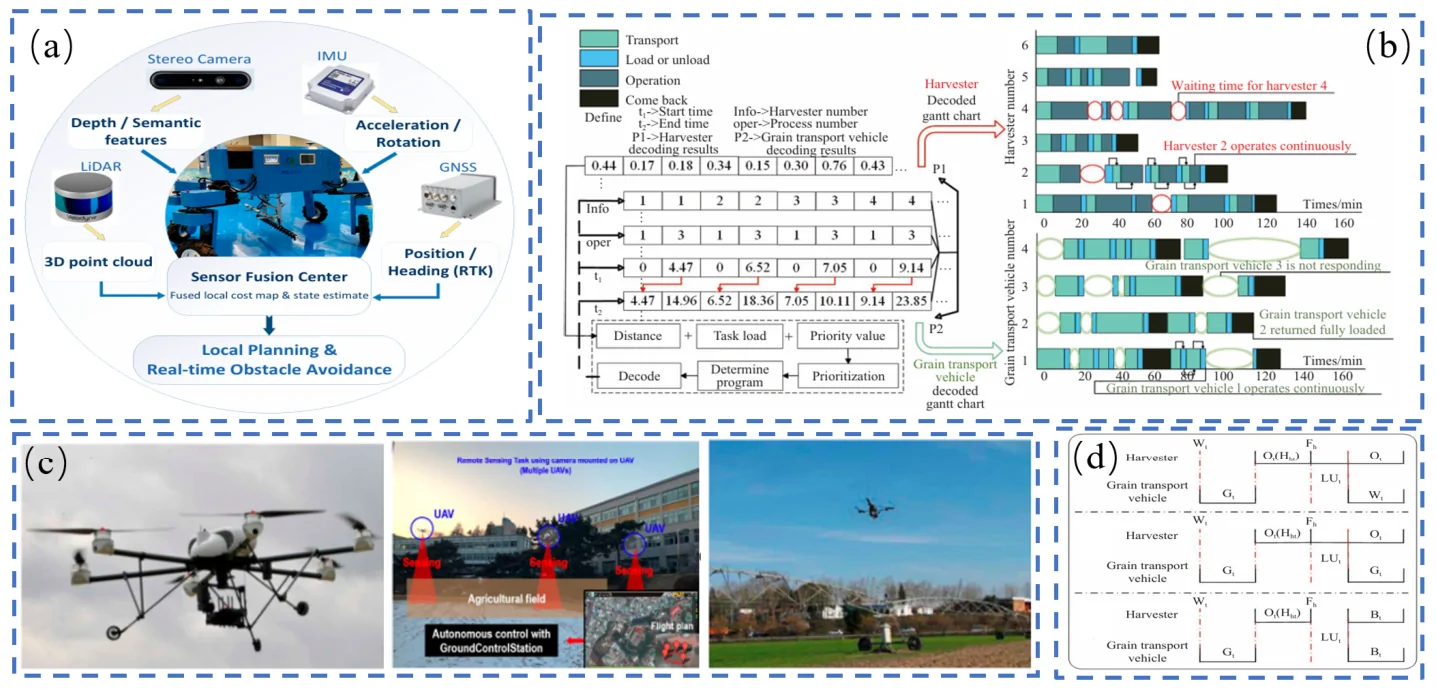
**Global operations research optimization and heuristic task allocation paradigms for agricultural harvesting-transport fleets.** (**a**) Multi-sensor Perception Pipeline for Agricultural Robot Navigation [17]. (**b**) Encoding and decoding [93]. (**c**) Examples of multi-UAV cooperative systems [107]. (**d**) Unloading process [93].

Finally, narrowing the focus from vast open fields to the closed environments of intelligent greenhouses, where physical space is extremely constrained and multi-stage operational processes are highly coupled, the optimization accuracy and parallel efficiency of algorithms face even more extreme tests. Addressing the high-density 3D logistics bottleneck in greenhouses, Zhao et al. constructed a GMRTS mathematical operations research model with the strict minimization of system makespan as the sole convergence objective, and tailored a Hybrid Attraction-Repulsion Optimization Algorithm (HAROA) for it [106]. This underlying algorithm cleverly draws inspiration from fluid mechanics and physical field theory: during the initial global search, it utilizes an eddy diffusion mechanism to construct an initial solution set with high diversity; in the mid-stage, it employs an attraction convergence mechanism to drive the algorithm toward high-potential solution regions; and in the late search stage, where algorithms are prone to stagnation, it triggers a turbulent transition mechanism to ensure the algorithm successfully escapes the computational trap of local optima with high probability. Multi-dimensional benchmark comparative experiments conclusively confirm that when facing extreme task scheduling with high concurrency and high-dimensional constraints in greenhouses, this physically heuristic strategy exhibits an overwhelming “dimensionality-reduction” advantage over many traditional meta-heuristic architectures in terms of solution precision limits and long-period convergence stability. The global operations research optimization and heuristic task allocation paradigms for these agricultural fleets are systematically summarized in Figure 3. This includes the multi-sensor perception pipeline (Figure 3a) and the complex encoding and decoding processes (Figure 3b) essential for global routing. Additionally, concrete examples of multi-UAV cooperative systems and precise unloading processes are presented in Figure 3c,d, validating the overwhelming advantage of these advanced heuristic strategies in resolving high-dimensional constraints.

### 4.2. Data-Driven Feedforward Predictive Scheduling

As the dimension of infield logistics in modern agriculture expands exponentially, high-frequency dynamic interaction between heterogeneous fleets has become the system norm. A critical horizontal comparison between traditional operations research (OR) and emerging data-driven methods highlights distinct methodological bottlenecks in both paradigms. While traditional static OR models excel in pursuing global theoretical optima, they suffer from a severe flaw: unacceptable computational latency that renders them brittle against sudden physical disturbances like dynamic obstacles or sudden yield fluctuations [88]. Conversely, while data-driven algorithms (e.g., MARL) resolve this latency by shifting computation to the training phase, they introduce a new, formidable bottleneck: the ‘Sim-to-Real’ gap. Models trained in idealized simulations often fail unpredictably under the stochastic, nonlinear dynamics of real-world unstructured farmland, marking a clear research gap in the robust generalization of learning-based scheduling algorithms.

Regarding the dimension of advanced prediction and global coordination for macro-fleet routing, decentralized dynamic architectures with high fault tolerance are gradually replacing traditional centralized instruction allocation mechanisms. Lujak et al. prospectively focused on the challenges of wide-area, large-scale fleet collaboration across multiple fields and owners, constructing a rigorous mathematical model for the Agriculture Fleet Vehicle Routing Problem (AF-VRP) [12]. Starting from the underlying logic of systems engineering, this research explicitly points out that highly autonomous Agricultural Mobile Robot (AMR) clusters must thoroughly break the inherent bottlenecks of traditional centralized control architectures in terms of wide-area scalability and central computational limits. By establishing decentralized dynamic task-adaptive allocation mechanisms at the bottom layer [108], the system successfully realizes high-fidelity advance prediction of unknown potential logistics operation demands, as well as dynamic flexible coordination of heterogeneous transport capacity resources on a global scale [109].

Shifting focus to real-time path prediction and trajectory evolution in unknown, highly dynamic environments, Reinforcement Learning (RL) frameworks have demonstrated the potential for feedforward decision-making that transcends traditional analytical algorithms [110,111]. In exploring the intelligent path planning challenge for agricultural robots traversing complex, highly interfered farmland, Yang et al. developed an end-to-end autonomous decision-making hub based on Deep Reinforcement Learning (DRL) [19]. The breakthrough of this research lies in endowing agricultural robot networks with the ability to autonomously and implicitly learn optimal obstacle avoidance strategies and smooth path generation flows through high-frequency trial-and-error interactions with the environment in completely unknown, dynamic, unstructured farmland, thereby thoroughly decoupling the bottom-level logic from the heavy reliance on global, absolute, high-precision prior maps inherent in traditional navigation scheduling systems. On this theoretical basis, to fundamentally resolve the “Curse of Dimensionality” easily triggered by high-concurrency multi-machine scheduling, Zhao et al. innovatively introduced Multi-Agent Reinforcement Learning (MARL) architectures into collaborative path optimization and formation-following tasks for Multi-UAV systems in 3D continuous space [21]. Such purely data-driven, decentralized neural networks allow agent clusters to naturally emerge with highly tacit collaborative collision-avoidance and deadlock-prevention capabilities through distributed high-frequency interactions at the bottom layer [26,112]. The fact that this system can maintain highly coordinated cluster formation behaviors while thoroughly stripping away the global unified instruction center provides a highly valuable cross-domain theoretical reference and engineering mapping for heterogeneous heavy-duty ground agricultural machinery fleets executing extremely complex feedforward predictive scheduling.

The perfect blueprint of macro-level feedforward predictive coordination is inevitably and heavily dependent on high-frequency, high-precision bottom-level predictive control strategies at the micro-execution layer to provide a safeguard. Focusing on autonomous tractor systems with complex articulated trailer structures, Kayacan et al. developed an innovative distributed Nonlinear Model Predictive Control (DiNMPC) bottom-level execution framework [94]. This research cleverly fuses frontier Non-linear Moving Horizon Estimation (NMHE) technology, enabling the control system to possess high-fidelity advanced prediction of trailer future-horizon control inputs and high-frequency real-time measurement updates of system states, even when directly facing extremely complex articulated non-linear multi-body dynamics evolution and severe wheel slip interference caused by unpaved farmland surfaces. Physical field experiments conclusively demonstrate that this edge-side feedforward control architecture, while effectively stripping away the redundant computational burden of multi-machine collaboration, successfully suppressed the Euclidean tracking error limit of tractor-trailer combinations to approximately 3 cm during high-speed operating conditions, providing an extremely robust underlying dynamic guarantee for the micro-level safety landing of macro-scheduling [113].

**Figure 4:**
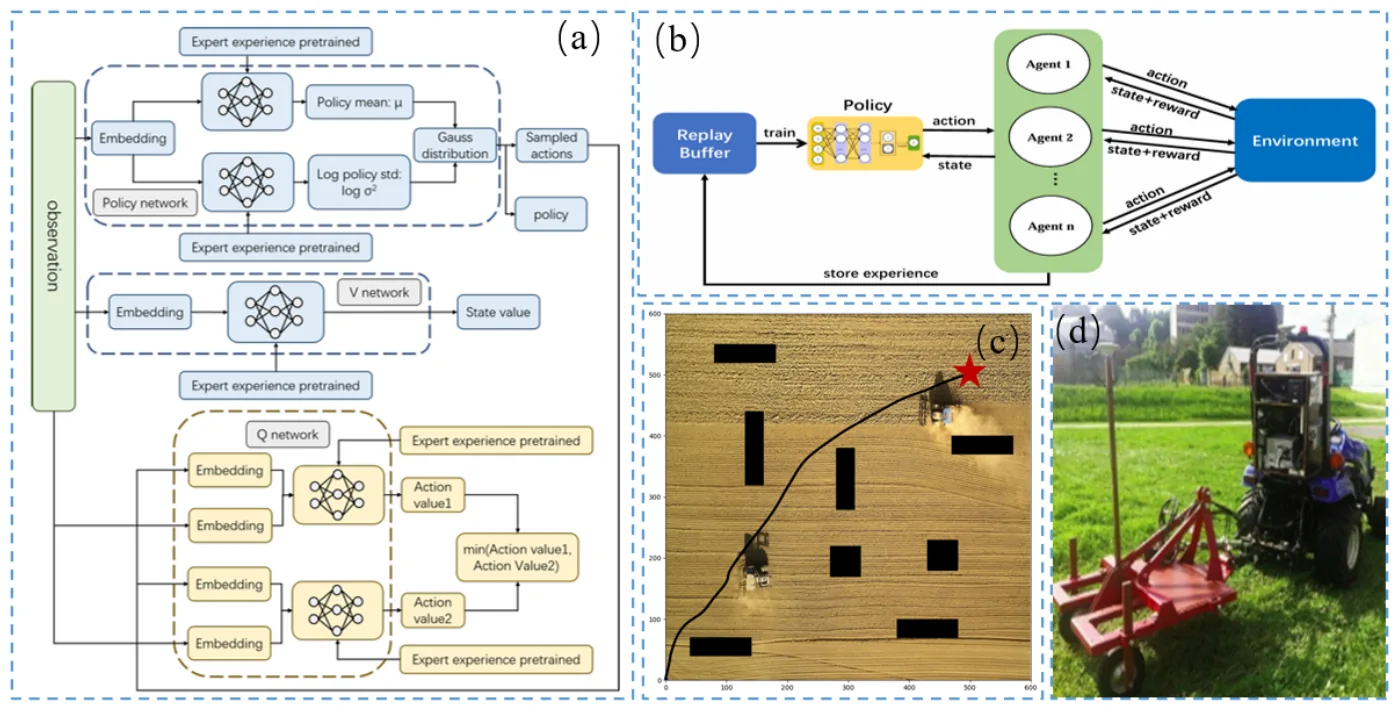
**Data-driven feedforward prediction scheduling and control paradigms in unstructured agricultural environments.** (**a**) The training process of the R-SAC algorithm [19]. (**b**) Model architecture of the path planning and following algorithm based on the multi-agent soft actor–critic framework [21]. (**c**) The planned path with target point of (500, 500) and dynamic obstacles [19]. (**d**) NMPC feedforward closed-loop for articulated agricultural machinery [116].

Furthermore, when facing dynamic physical obstacles with strongly random distributions in complex farmland (such as shuttling harvesting workers or intersecting heterogeneous agricultural machines), Wang et al. [114,115] creatively constructed a perception-driven obstacle-avoidance framework that strictly binds sensor observation arrays to learning algorithms [18]. By utilizing high-frame-rate LiDAR and visual tracking sensors to extract the spatiotemporal trajectories of dynamic obstacles, the system achieves precise state representation of motion velocities and directional vectors at the feedforward perception end. This high-fidelity sensory data acts as the direct input for neural network models, translating raw environmental perception into continuous control weights for local navigation. This demonstrates that the success of data-driven scheduling is fundamentally anchored in the accuracy and resolution of the onboard sensing suite. This underlying feedforward strategy, strongly coupled with data-driven and physical kinematics, endows agricultural robots with “predictive” active risk avoidance and “early detour” capabilities. Empirical data confirm that this hybrid strategy significantly boosts the success rate of local obstacle avoidance to over 90% in highly complex dynamic interaction environments, thereby thoroughly dismantling the technical limitations of the traditional, purely geometric DWA framework, which relies heavily on prior manual parameter tuning and is extremely prone to falling into local spatial deadlocks (local minima) within narrowly constrained spaces. This data-driven feedforward prediction scheduling and control paradigm in unstructured environments is comprehensively depicted in Figure 4. Figure 4a,b illustrate the training process of the R-SAC algorithm and the model architecture of the path planning algorithm based on the multi-agent soft actor-critic framework. Consequently, Figure 4c,d display the planned path circumventing dynamic obstacles and the NMPC feedforward closed-loop for articulated agricultural machinery, demonstrating how these hybrid strategies successfully dismantle the technical limitations of traditional frameworks.

### 4.3. Micro-Kinematic Constraints and Conflict Resolution

Regardless of how perfectly the global blueprint of macro-scheduling and the theoretical trajectories of feedforward prediction are deduced in virtual computational space, their physical closed loop must inevitably be safely realized through the deep resolution of underlying stringent kinematic constraints and high-frequency local multi-machine collision-avoidance strategies. Since modern heavy-duty agricultural machinery typically carries large-sized implements or presents complex multi-degree-of-freedom articulated physical structures, and given that real-world farmland physical workspaces (such as the extremely narrow row spacing in dense orchards or irregular headland turning areas) exhibit extreme geometric topological constraints, how to absolutely guarantee the physical interference safety and continuous trajectory smoothness of multi-machine clusters at the micro-execution level within such rigid spatial boundaries has become the core engineering difficulty in thoroughly bridging the “last mile” of farmland cluster collaborative scheduling [117]. In view of this, this section descends to the underlying control foundation [118], systematically elaborating from three micro-execution levels: restricted trajectory planning for complex long-sized implement mounting, high-precision collaborative tracking for master–slave formation operations, and multi-agent distributed spatial deadlock resolution.

In addressing the micro-trajectory planning challenge where complex implement-carrying entities interweave with extremely narrow spaces, traditional algorithms based on pure geometric search often fall into extreme trajectory conservatism due to the use of crude, single rectangular rigid-body expansion models, which in turn leads to a severe waste of available passable space. Facing the engineering pain point of agricultural traction vehicles carrying long-sized non-holonomic implements performing extreme turns in narrow headland zones, Wei et al. proposed a cascade trajectory planner that deeply balances physical safety with spatial utilization efficiency [63]. At the algorithmic front-end, this team innovatively introduced a high-frequency collision detection mechanism based on enveloping circles, thereby significantly accelerating the global exploration convergence efficiency of the Hybrid A* algorithm in complex configuration spaces. At the back-end, the researchers cleverly utilized the properties of differential flatness theory and multiple dynamic safety corridors mechanisms to achieve a perfect analytical decoupling of the strongly coupled kinematics of the main tractor body and the rear-mounted implement in the spatial manifold. This strategy successfully generated smooth, continuous trajectories that strictly satisfy non-holonomic multi-body vehicle dynamics constraints within the extremely constrained topology of orchard headlands.

Furthermore, shifting the focus to spatial navigation challenges with even more extreme narrow geometric boundaries, such as in greenhouses, Kulathunga et al. designed and deployed a highly modular, pure perception-driven underlying navigation framework. This research strictly integrated a multi-step progressive 3D point cloud processing pipeline at the front-end to execute high-fidelity crop physical boundary line estimation and raw trajectory refinement, while innovatively introducing a curvature-adaptive adjustment mechanism into the Pure Pursuit lateral controller at the back-end [119]. Real-world, in situ tests show that this closed-loop control system achieved astonishingly high lateral control precision in a real strawberry plastic greenhouse with extremely tight row spacing—the path average tracking deviation was pushed down to a limit of only 0.08 m, thereby thoroughly ensuring the collision-free, safe passage of the agricultural robot platform between extremely narrow crop rows at the most microscopic physical execution scale [116].

Turning the focus to the dimension of high-precision collaborative control in master–slave (Leader–Follower) formations, continuous-flow harvesting operations in open fields inevitably require multiple heavy agricultural machines to execute extremely stringent, ultra-close-range dynamic platooning and real-time relay in physical space [120]. Focusing on the dynamic collaborative grain-unloading problem between autonomous tractor-trailer systems with strong model uncertainties and combine harvesters, Shojaei deeply explored and constructed a high-performance collaborative tracking control base based on neuro-adaptive Proportional–Integral–Derivative (PID) control [95]. By introducing complex non-linear coordinate transformations and Prescribed Performance control theory, this architecture provides consistency guarantees at the mathematical level [121]: it not only enables the follower tractor to precisely lock onto the desired relative longitudinal/lateral spacing and heading angle with extremely low error when following the main harvester, but also thoroughly blocks the physical interference risks easily occurring between heavy-duty equipment through the underlying built-in dynamic anti-collision constraint mechanism. Pushing the robustness boundaries in this field further, Lu et al. proposed a master–slave robust control framework with strong external anti-interference capabilities specifically for dynamic collaborative scenarios involving harvesters and tractor-trailers in farmland environments with restricted spatial topologies [122]. This research cleverly fused robust Sliding Mode Control (SMC) with Radial Basis Function (RBF) neural networks at the algorithmic level [123]. This non-linear coupling strategy not only perfectly compensates for the extremely complex strong non-linear model uncertainties underlying the multi-body articulated control system through the online approximation capability of neural networks, but also seamlessly embedded an improved Artificial Potential Field (APF) method into the control closed loop [124], thereby ensuring that the two heterogeneous “giants” can still achieve millisecond-level efficient autonomous obstacle avoidance when executing approaching relay and collaborative unloading operations within extremely narrow spaces.

**Figure 5:**
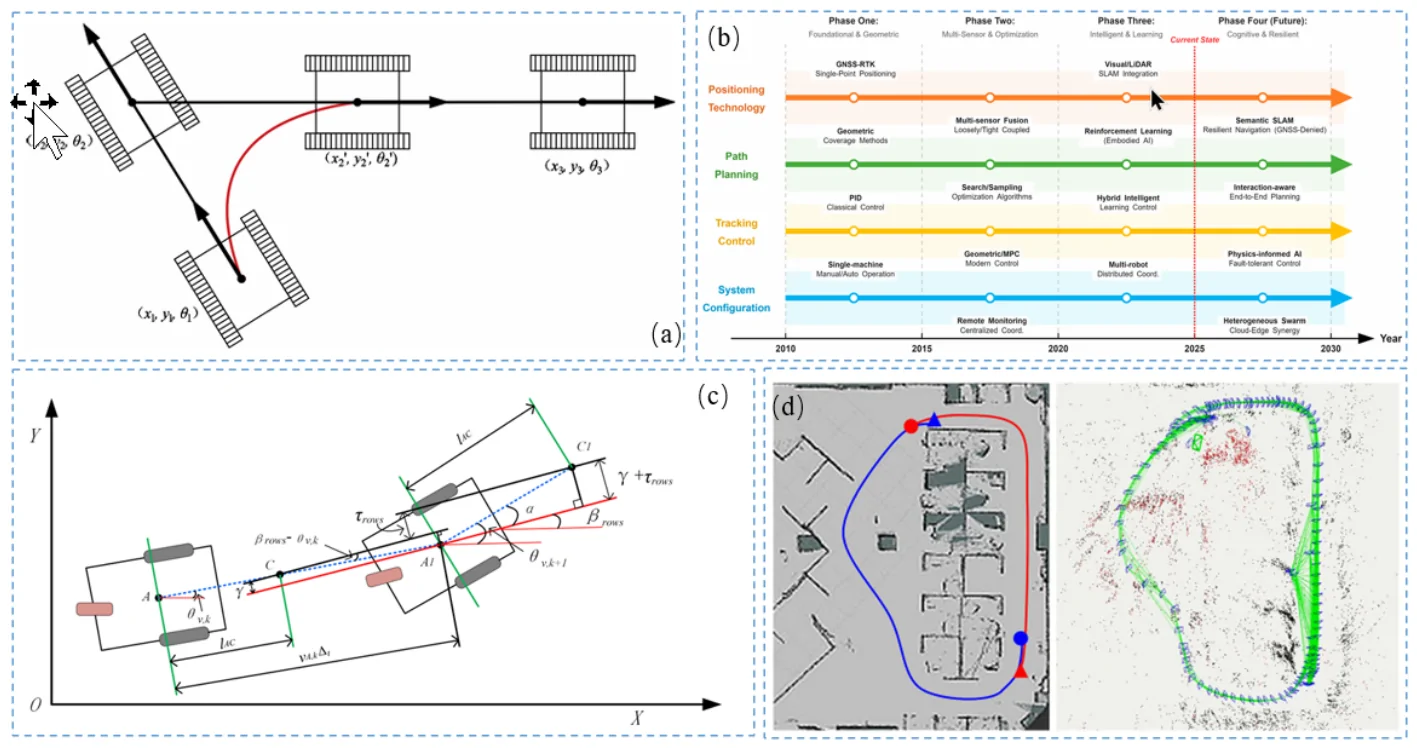
**Underlying kinematic constraints and high-frequency conflict resolution for agricultural mobile robots in complex habitats.** (**a**) Geometric modeling of microscopic trajectory deviation considering non-holonomic dynamic hard constraints [62]. (**b**) Error model of underlying trajectory tracking lateral controllers to overcome extreme physical boundaries [17]. (**c**) Vector analysis of active collision avoidance and posture reconstruction by underlying control systems [36]. (**d**) Measured results of distributed multi-agent collaborative mapping and local deadlock self-resolution isolated from global absolute positioning [126].

However, when large-scale intelligent agricultural machinery fleets converge at high frequency within highly unstructured and intricate farmland road networks, multi-agent systems are highly susceptible to trajectory conflicts and irreversible deadlock states within local physical spaces. To comprehensively elevate the micro-level motion maneuverability and spatial conflict self-resolution capability of fleets in unstructured environments, Ait Dahmad et al. were the first internationally to propose the deep integration of Interval Type-2 fuzzy logic control strategies with underlying behavioral response navigation frameworks for omnidirectional agricultural mobile robot platforms featuring four-wheel drive and four-wheel independent steering (4WD/4WS) [64]. This underlying flexible control paradigm, oriented toward uncertainty inference, endows agricultural machinery platforms with smoother high-frequency trajectory correction and pose reconstruction capabilities when facing highly dynamic obstacles and extremely constrained physical boundaries [125], thereby significantly reducing the local physical deadlock risk for multi-machine close-proximity interactions in a probabilistic sense. Furthermore, in large-scale wide-area cluster scheduling scenarios lacking stable global absolute positioning (e.g., GPS-denied), breaking the reliance on centralized pose servers has become another major challenge for deadlock resolution. To address this, Zhang et al. creatively designed and deployed a fully distributed multi-robot monocular visual Simultaneous Localization and Mapping (SLAM) underlying architecture for large-scale, completely unknown farmland environments. This collaborative perception system thoroughly discards the reliance on prior knowledge of the fleet’s relative poses; instead, it performs highly robust scene recognition independently on the local machine by extracting high-dimensional visual features and combining them with Bag-of-Words (BoW) image high-frequency matching algorithms. More ingeniously, when the underlying system detects severe trajectory tracking loss or high-probability collision conflicts in multi-machine spatiotemporal trajectories, the algorithm immediately triggers an active loop closure intervention mechanism [126], forcing the robot to proactively reconstruct and deflect its predetermined motion trajectory to search for historical visual loop closures and fundamentally eliminate accumulated odometry drift. This frontier control strategy, which fuses active optimization with distributed perception, enables multi-robot collaborative clusters to autonomously implement seamless stitching of local topological maps and efficient resolution of local multi-machine spatial conflicts at the bottom layer, even under extremely isolated conditions completely free from the intervention of central computing servers. The resolution of these underlying kinematic constraints and high-frequency conflicts for agricultural mobile robots is visualized in Figure 5. Figure 5a,b detail the geometric modeling of microscopic trajectory deviations and the error model of underlying lateral controllers designed to overcome extreme physical boundaries. Furthermore, Figure 5c,d illustrate the vector analysis of active collision avoidance and the measured results of distributed multi-agent collaborative mapping, proving the system’s robust capability to execute local deadlock self-resolution autonomously.

## 5. Scene Practice: Spatiotemporal Collaborative Operational Paradigms in Typical Farmland

Regardless of how acute the multi-modal perception network of the underlying digital foundation may be, or how exquisite the evolution and deduction of the central feedforward prediction and operations research scheduling algorithms within the virtual digital twin space are, the final validation closed-loop of their theoretical value and engineering robustness must necessarily and inevitably land in the real-world farmland environment, which is highly dynamic, unstructured, and permeated with stochastic physical disturbances. The agricultural “harvest–transport” cluster logistics system is never a homogenized solution paradigm with universal applicability. Distinct crop varieties, highly diverse agronomic planting patterns, and rigorous topological space constraints endow different farmland logistics systems with highly heterogeneous spatiotemporal evolutionary dynamics. Therefore, the ultimate key to thoroughly bridging the full software-hardware chain of Agricultural Cyber-Physical Systems (ACPS) lies in how to deeply couple multi-agent collaboration and control theories—which often remain under laboratory ideal assumptions—with the unstructured physical boundaries of specific scenarios, and adapt them to engineering practices that can substantially enhance large-scale productivity.

Based on this, this section shifts the research perspective from abstract operations research deduction and mathematical logic down to the concrete physical forms of agricultural habitats, systematically reviewing and dissecting frontier implementation paradigms of multi-robot collaborative clusters in three most representative complex farmland environments. The subsequent content will sequentially focus on and delve into the following:

“Continuous-flow” Collaboration in Field Crop Scenarios: Focusing on expansive field environments with open physical boundaries and high operational standardization, we deeply analyze how heavy-duty combine harvesters and relay grain-transport fleets realize high-speed, non-stop “unloading-on-the-go” and spatiotemporal collaborative unloading of large-volume continuous flows, relying on high-frequency macro–micro closed-loop algorithms.

“Discrete-flow” Scheduling in High-density Orchard Scenarios: Placed deep within unstructured orchards characterized by severe canopy GNSS occlusion and rugged terrain, we dissect how ground-based flexible transport AGVs effectively suppress long-distance cumulative mapping drift and break through the physical kinematic constraints of extremely narrow row spacing, thereby achieving discrete, refined point-to-point harvesting and high-frequency hub-and-spoke scheduling.

“3D High-density” Relay in Greenhouse Scenarios: Facing modern enclosed greenhouses where 3D topological space is extremely constrained yet characterized by high IoT digitization, we explore how high-payload mobile collaborative robots (CoBots) achieve highly trusted and physically safe Human–Robot Collaboration (HRC) with dense human harvesting workers, thereby constructing a cross-level, 3D, high-density digital logistics transfer network.

### 5.1. Open-Field Crops (Continuous Flow): High-Speed Dynamic Relay of Heavy-Duty Agricultural Machinery

Large-scale, standardized plain farmland (such as major grain-producing regions for wheat, corn, and soybeans) represents the typical agricultural habitat with the highest maturity of single-machine automation and the most expansive physical topological boundaries. During the concentrated harvest period for these staple crops, the cumulative filling rate of the grain bin on heavy-duty combine harvesters is extremely rapid due to the massive spatial yield base of field crops. Consequently, traditional passive discrete operational modes based on “static stopping for unloading after reaching full capacity” [127] inevitably lead to severe process interruptions and a sharp decline in overall spatiotemporal efficiency. In view of this, farmland logistics present highly strongly coupled “continuous flow” characteristics. This physical trait imposes the rigorous requirement that the logistics relay grain-transport fleet must possess the capability for precise side-by-side platooning and dynamic “unloading-on-the-go” while maintaining strict relative motion poses with the main harvester under high-speed mobile operating conditions.

Regarding the macro-system-level engineering value of continuous-flow collaborative operations in open fields, Wei et al. provided a systematic, panoramic review of top-level planning technologies, underlying communication networking, and non-linear motion control for multi-agent agricultural machinery collaboration. This study explicitly reveals that when facing large-scale, high-intensity, time-critical harvesting tasks with extremely urgent time windows, high-fidelity spatiotemporal collaborative mapping between heavy-duty harvesters and grain-transport relay vehicles is no longer merely an optimization measure to reduce the high idle rate of agricultural machinery assets. More fundamentally, it constitutes essential, “hardcore” engineering support for hedging against the structural impact of global agricultural labor aging at the macro level and absolutely guaranteeing the security of the underlying supply chain for bulk agricultural products [16].

To ensure that the aforementioned macro-collaborative potential is effectively transformed into physical productivity in real unstructured open-field scenarios, system algorithms must fundamentally conquer the high-dimensional matching problem between “heterogeneous transport supply and dynamic spatial yield” during high-speed mobile interaction. Addressing the systemic pain points of sluggish scheduling response and high non-productive costs in traditional field collaborative harvest–transport operations, Liu et al. fully imported real, complex farmland geometric boundaries and multi-source heterogeneous parameters of the fleet into a multi-objective joint scheduling model. The team innovatively proposed and deployed an Improved Marine Predators Algorithm (IMPA) [93]. This underlying algorithm cleverly fuses radial transformation matrix topology with high-frequency dynamic search mechanisms, thereby drastically elevating the algorithm’s global rapid optimization capability and its ability to escape local extrema in high-dimensional constrained search spaces. Relying on dual-mapping verification via high-fidelity simulation and actual field habitats, this optimization strategy can dynamically coordinate combine harvesters with different payload limits and relay grain-transport fleets with extreme efficiency. Under the rigorous prerequisite of absolutely ensuring that the main harvester executes “continuous-flow” operations without deceleration or interruption, it successfully achieved the dual absolute minimization of system-level global scheduling compensation costs and heterogeneous fleet idle waiting times, thereby fortifying an indestructible spatiotemporal operations research foundation for extreme logistics scheduling of continuous field flows [128].

**Figure 6:**
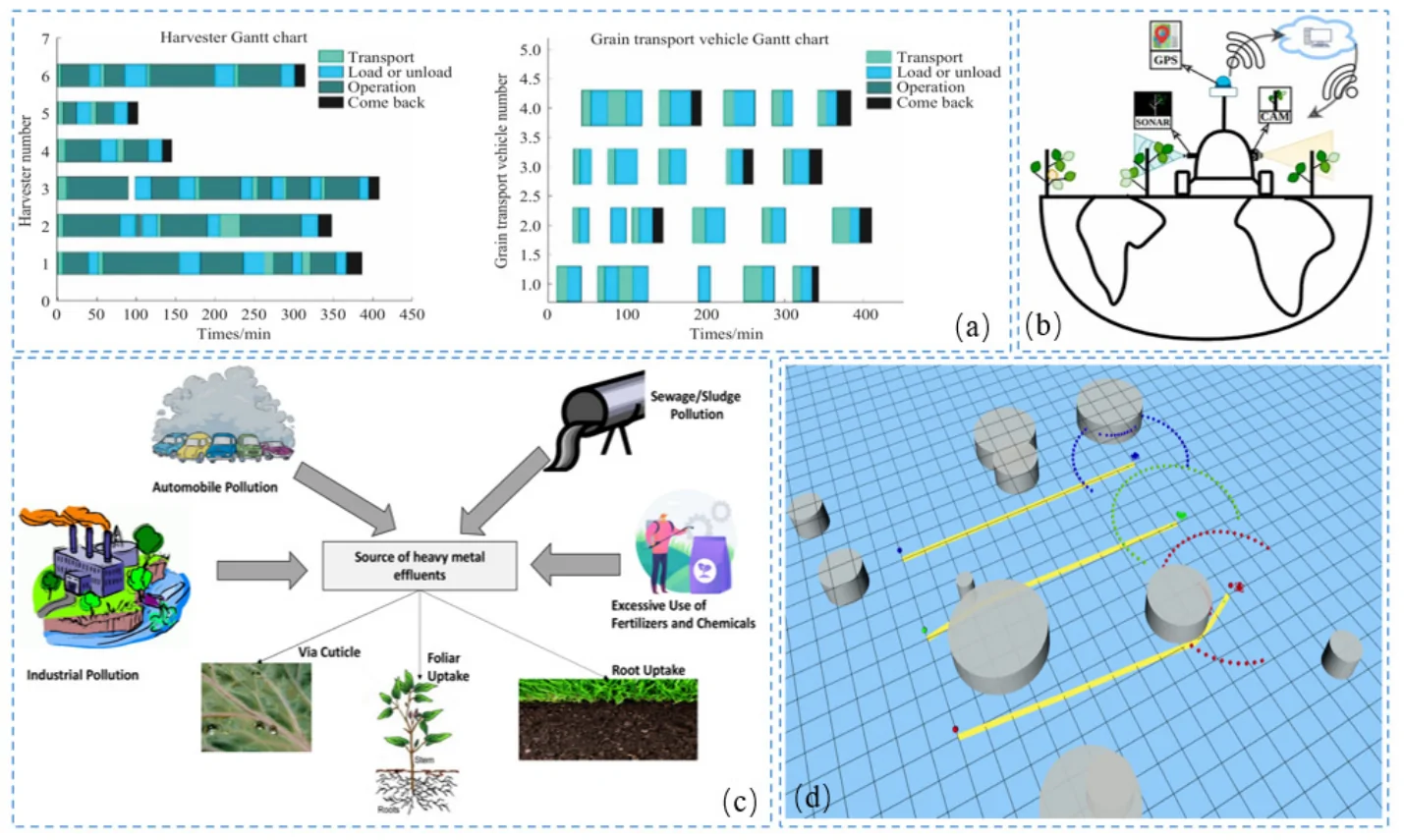
**Dynamic transfer and spatiotemporal collaborative operation paradigms of heavy agricultural machinery in open-field continuous flow harvesting scenarios.** (**a**) Optimal routing and Gantt charts for cooperative scheduling of harvesters and grain carts under hard time-window constraints [93]. (**b**) Closed-loop navigation control system topology for heavy agricultural machinery adapted to complex field conditions [116]. (**c**) Simulation of dynamic platooning and high-precision formation-following trajectories based on MARL [43]. (**d**) Distributed edge computing and sensor network architecture supporting wide-area high-frequency communication in open fields [21].

Furthermore, the efficient operation of a continuous-flow field logistics system is by no means confined to resource coordination at the macro-algorithmic level; it constitutes an extreme engineering challenge concerning underlying dynamics and micro-execution control. Executing side-by-side dynamic unloading during high-speed movement harshly requires two heavy heterogeneous agricultural machines weighing tens of tons to maintain extremely high-precision lateral and longitudinal relative topological spacing in 3D continuous space in real-time. Even more severely, for grain-transport tractors towing heavy trailers, the random undulations of non-paved field terrain and the spatiotemporal fluctuations of soil mechanical properties easily induce severe sideslip or non-linear yaw in articulated trailers, thereby dramatically pushing up the systemic risk of physical interference and catastrophic collisions between multiple machines. Focusing on this micro-control pain point, Shojaei conducted an in-depth exploration of non-linear control theory regarding the dynamic collaborative grain-unloading problem between autonomous tractor-trailer systems and combine harvesters under strong model uncertainty boundaries in field habitats [95]. This study innovatively constructed a high-order intelligent tracking control base based on Neuro-Adaptive Proportional–Integral–Derivative (PID) control. In the underlying algorithmic deduction, this control architecture cleverly utilizes complex non-linear coordinate transformation topologies and Prescribed Performance Function constraints to perform extremely rigorous closed-loop compensation for non-holonomic kinematic errors of multi-body articulated vehicles under time-varying poses. Actual physical test results conclusively indicate that this flexible control system not only enables the follower tractor to precisely lock onto the desired relative yaw angle and relay spatial distance with extremely low steady-state error when following the main harvester but also significantly suppresses the strong non-linear external physical disturbances excited by muddy farmland surface morphologies [129]. This breakthrough in underlying control strategy fundamentally constructs an extremely robust system dynamics defense line for the micro-space interaction and absolute physical safety of heavy-duty heterogeneous agricultural machinery under extremely high-throughput, continuous-flow operating conditions. Figure 6 systematically visualizes the dynamic transfer and spatiotemporal collaborative operation paradigms of heavy machinery in open-field continuous flow scenarios. The optimal routing and Gantt charts for cooperative scheduling under hard time-window constraints are presented in Figure 6a. To support this macro-scheduling, Figure 6b–d demonstrate the closed-loop navigation control topology, MARL-based dynamic platooning simulations, and the distributed edge computing architecture, jointly constructing an extremely robust defense line for physical safety under high-throughput conditions.

### 5.2. High-Density Orchards (Discrete Flow): Narrow-Row Shuttling and Hub-and-Spoke Scheduling

Unlike the robust, continuous-throughput operational paradigm found in open-field habitats, the “harvest–transport” collaborative system in modern high-density orchards (such as standardized apple or citrus plantations) exhibits highly heterogeneous “discrete flow” topological characteristics. Constrained by the extreme non-uniformity of fruit spatial distribution and the rigorous precision requirements for non-destructive end-effector picking [130,131], orchard macro-logistics must inevitably evolve into high-frequency, point-to-point discrete material transfers between multiple specialized picking robots [132] and highly flexible transport AGVs at specific topological nodes (e.g., extremely narrow inter-row spaces beneath tree canopies or collection hubs at field edges). However, the complex 3D canopy structure of orchards not only triggers severe attenuation or even long-term denial of global GNSS signals (GPS-denied), but the extremely rugged, unpaved surface terrain, multi-path illumination variations that fluctuate wildly with seasons and meteorological conditions, and constrained physical row spacing collectively construct an extremely high environmental resistance barrier [133]. This poses severe control challenges for the long-term high-dimensional stable shuttling of clusters within the logistics network and for precise docking at discrete nodes.

In the depths of an orchard where global absolute positioning priors are completely stripped away, constructing a high-fidelity 3D digital map that possesses global topological consistency and is highly adaptive to undulating micro-terrain is the physical cornerstone for anchoring discrete logistics scheduling nodes [134,135,136]. Addressing the core pain point that mobile robot platforms are highly susceptible to cumulative mapping drift during long-term operation in large-scale orchard habitats, Liang et al. independently developed and deployed a multi-source heterogeneous sensor fusion hardware architecture with high time-synchronization precision [137]. Their research deeply integrated and improved the FAST-LIO2 high-frequency odometry framework in the back-end algorithm; by relying on robust active loop-closure detection mechanisms and global factor graph joint optimization algorithms, they fundamentally eliminated multi-dimensional cumulative pose errors in the temporal matching process of laser point clouds. This laid a solid foundation of high-precision prior maps for underlying transport AGVs to execute flexible shuttling among the complex, unstructured obstacles of the orchard and achieve centimeter-level precision docking at multi-level collection hubs. After breaking through the bottleneck of relative pose estimation, the chassis’s physical adaptability to unpaved micro-terrain immediately ascends to another system-level core of scheduling execution [138]. Relying on the optimized LeGO-LOAM algorithm to complete the basic 3D point cloud reconstruction of the orchard, Cao et al. innovatively introduced Bayesian Generalized Kernel (BGK) inference technology to directly map and generate continuous “traversability maps” representing physical resistance on the point cloud surface [139]. In the underlying control loop, the team utilized Statistical Outlier Removal (SOR) filtering technology to decouple and reduce the dimensionality of high-dimensional environmental features, and deeply embedded the RRT* (Rapidly exploring Random Tree Star) algorithm. In the local control layer of fully loaded transport AGVs, this realized proactive perception and flexible obstacle avoidance for high-risk terrain, thoroughly blocking the systemic risks of bogging down—such as chassis grounding or rollover—when heavy agricultural machinery traverses extremely muddy or steep, pockmarked terrain [137,140,141].

Focusing on the orchard-specific scenarios of shuttling through extremely narrow canopy rows and high-concurrency micro-level obstacle avoidance, the deep closed-loop integration of multi-modal heterogeneous perception and underlying non-holonomic kinematic constraints plays a decisive role. Addressing the characteristics of high-frequency alternating mottled light and shadows and heavily cluttered branch and leaf occlusion in orchard habitats, Han et al. developed a high-dimensional feature extraction framework based on the deep fusion of LiDAR point clouds and 2D camera vision. This heterogeneous fusion system not only breaks through the physical field-of-view limitations of single sensors to precisely delineate the 3D envelope boundaries of intertwined dynamic and static obstacles within complex orchard rows, but also endows the robot with the capability to maintain extremely high underlying local positioning robustness and continuous navigation stability even when facing extreme confinement with intense backlight interference or severe canopy occlusion [36]. Built upon this perception foundation, and addressing the randomly distributed obstacles and strongly non-linear topological branch barriers within the highly unstructured orchard inner network, Ye et al. proposed and validated a Continuous Bi-directional Rapidly exploring Random Tree algorithm with strict kinematic hard constraints (CBQ-RRT) [62]. This underlying trajectory planner not only theoretically ensures that the feedforward output of the global obstacle-avoidance path strictly conforms to the non-holonomic multi-body dynamic limits of agricultural vehicles (such as the minimum curvature radius constraints of Ackerman steering), but also achieves rapid convergence and curvature smoothing in collision-free path searching within extremely high-dimensional solution spaces. This breakthrough in micro-control strategy significantly reduces the invalid compensation time consumed by heavy-duty transport equipment due to high-frequency start-stop and attitude reconstruction within extremely narrow physical channels, providing high-throughput underlying micro-execution guarantees for the orchard discrete-flow scheduling system.

**Figure 7:**
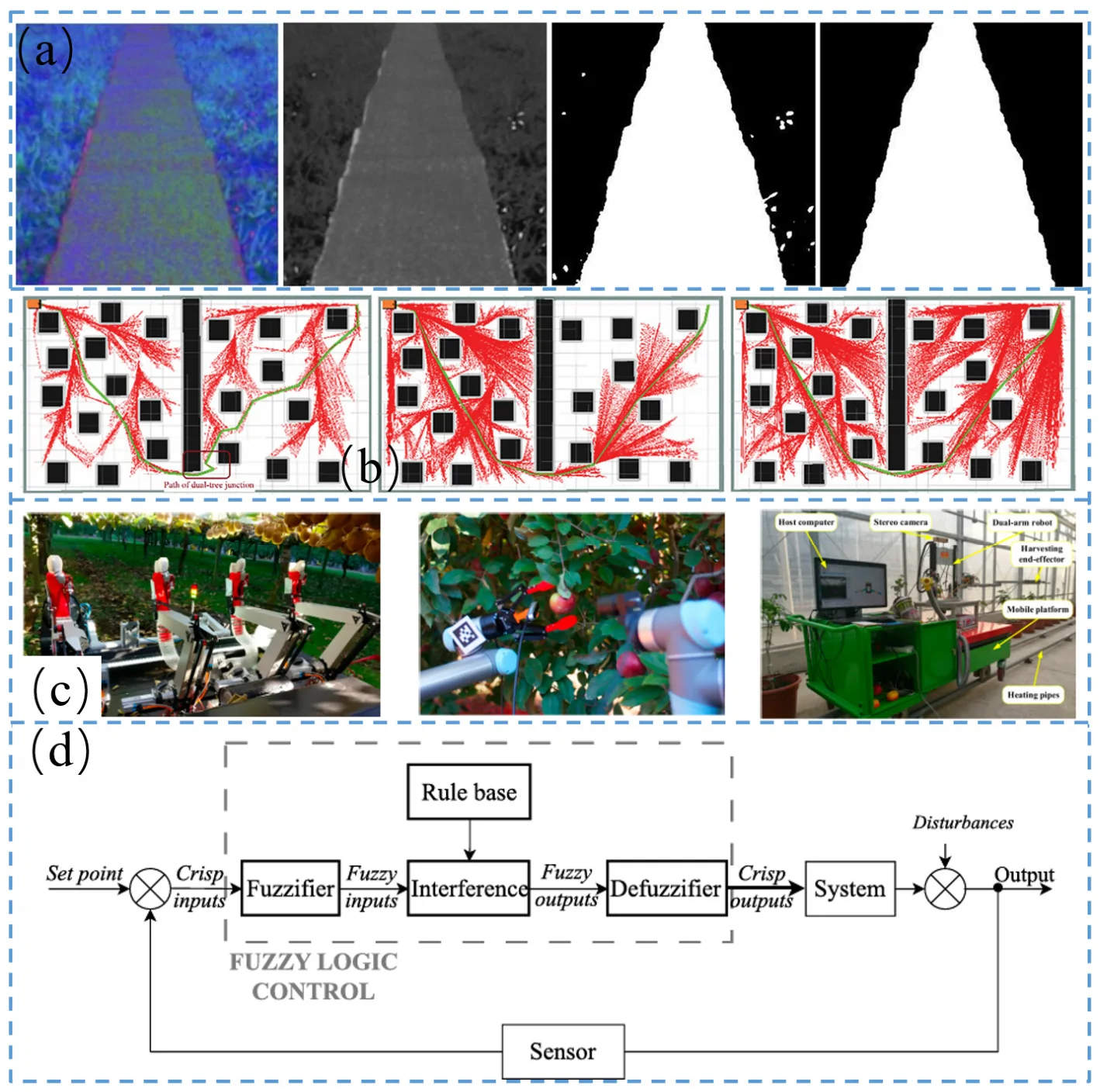
**Flexible shuttle and cooperative harvesting-transport paradigms without GNSS priors in dense orchard “discrete flow” scenarios.** (**a**) Local 3D point cloud mapping and tree trunk feature extraction based on LiDAR in heavily occluded GNSS-denied environments [36]. (**b**) Measured flexible path planning across irregular obstacles in complex unstructured orchard narrow-row habitats [62]. (**c**) Field validation of multi-robot cooperative operation (dual-arm harvesting and transport) in real vineyard/orchard environments [107]. (**d**) Underlying robust tracking and anti-slip compensation control mechanisms for rough orchard terrain and high-frequency slip disturbances [116].

Ultimately, the deep closed-loop of underlying multi-modal high-precision mapping, high-dimensional heterogeneous environmental perception, and non-holonomic micro-trajectory planning algorithms jointly construct the indispensable spatiotemporal topological and dynamic support for the orchard discrete-flow logistics collaborative scheduling network. This scheduling evolution paradigm of macro–micro deep coupling was rigorously theoretically verified in the systematic, panoramic analysis conducted by Lytridis et al. regarding heterogeneous collaborative robot systems in complex agricultural habitats [107]. This research explicitly reveals that through rigorous spatiotemporal decoupling of high-concurrency operational flows, the system deploys dual-arm (or multi-arm) collaborative harvesting actuators with high-redundancy kinematic degrees of freedom at the logistics link front-end, focusing on executing high-precision, non-destructive detachment and flexible grasping of unstructured target fruits [142,143]. Meanwhile, at the macro-transport back-end, it relies entirely on high-payload ground flexible transport AGVs equipped with the aforementioned high-order autonomous perception and underlying anti-deadlock navigation frameworks to execute large-volume, high-frequency relay transfers between complex orchard physical collection and distribution nodes. This perfect closed-loop and deep collaboration between “high-dimensional precision operation at the front-end” and “high-throughput flexible transport at the back-end” in the physical spatiotemporal dimension fundamentally and thoroughly dissolves the systemic resistance bottleneck of discrete-flow scheduling in complex, unstructured orchard habitats. This iconic engineering leap decisively declares that the modern orchard intelligent operation equipment system has fully dismantled the technical barriers of “individual isolated intelligence” and is irreversibly striding toward a substantial evolutionary era of “multi-machine highly heterogeneous collaboration and ubiquitous network interconnection.” The flexible shuttle and cooperative harvesting-transport paradigms in dense orchard ‘discrete flow’ scenarios without GNSS priors are presented in Figure 7. Figure 7a,b showcase the local 3D point cloud mapping in heavily occluded environments and the measured flexible path planning across irregular obstacles. Complementing this, Figure 7c,d display field validations of multi-robot cooperative operations and the underlying robust tracking mechanisms adapted for rough terrain, thoroughly dissolving the systemic resistance bottleneck of discrete-flow scheduling.

### 5.3. Vertical High-Density Greenhouses: Cross-Hierarchical Relay and Human–Robot Collaboration (HRC)

Modern smart greenhouses exhibit extreme spatial compactness and high-density three-dimensional cultivation characteristics [144], with the economic crops cultivated within them often possessing exceptionally high added value. Distinct from the relatively open topological boundaries of field environments or standard orchards, the logistics corridors within greenhouses feature extremely narrow and constrained characteristics. Furthermore, during operational cycles, it is inevitable that intelligent agricultural machinery and high-density human labor must share the same narrow physical workspace within the same spatiotemporal dimension. Given that current fully autonomous flexible robotic arms still face insurmountable engineering bottlenecks in end-effector harvesting success rates and large-scale deployment costs when confronted with highly unstructured canopy occlusion [145], cluster scheduling within greenhouses is undergoing a profound shift toward hybrid logistics paradigms characterized by cross-hierarchical heterogeneous relay and Human–Robot Collaboration (HRC) [146].

Focusing on the top-level design and social-cognitive dimensions of HRC synergy mechanisms, Yerebakan and Hu provided a panoramic system review of human–machine interaction architectures in modern agricultural habitats [147]. Their research explicitly points out that deploying multi-agent robotic clusters with high endurance and heavy payload capacities as foundational mobile collection and transport bases, while seamlessly integrating the unstructured precision manipulation and high-level cognitive decision-making capabilities demonstrated by human workers at the frontline, represents the most practical and economically efficient scheduling topology for breaking the current greenhouse logistics stalemate. Following this collaborative logic, Vasconez et al. further analyzed the micro-boundaries of dynamic interaction, emphasizing that when heterogeneous transport AGVs execute high-frequency concurrent operations with human workers within extremely restricted corridors, the system must embed rigorous Human–Robot Interaction (HRI) hard-boundary collision-avoidance safety mechanisms into the control layer [148]. Moreover, there is an urgent need to develop high-level kinematic planning algorithms equipped with human-intention awareness (such as adaptive active following and topologically safe overtaking) to completely eliminate physical interference and comprehensively enhance the spatiotemporal fluency of global scheduling [149]. Furthermore, transcending pure physical kinematic constraints, Benos et al. forward-lookingly emphasized that the design of high-level collaborative system architectures must extend into social and engineering psychology dimensions, deeply considering physical ergonomics boundaries and the cognitive computational load of workers in complex operational environments, thereby fundamentally fortifying the system’s perceptual safety defenses and the synergistic trust of human operators during practical engineering deployment [11].

To ensure that the aforementioned grand collaborative logistics vision is translated into actual productivity at the micro-physical execution level, the underlying transport robot platforms must first overcome the prerequisite challenge of high-frequency, high-precision spatial anchoring of highly dynamic human workers within topologically complex plastic greenhouse networks and three-dimensional high-density crop barriers. Addressing this core positioning pain point, Polvara et al. creatively constructed and validated a cutting-edge active perception control framework named Navigate-and-Seek [60]. This core algorithm deeply incorporates a Topological Particle Filter (TPF) adapted to nonlinear environments and tightly couples it with a Next-Best-Sense (NBS) anticipatory optimization planning strategy. Driven by this mechanism, the robotic platform can rely on the strong coupled perception flow of multi-source heterogeneous sensors (deep fusion of LiDAR point clouds, RFID radio frequency signals, and ubiquitous IoT GNSS data) to execute active spatial exploration within unknown greenhouse stereotactic topological road networks, and lock onto the absolute spatial coordinates of dynamic harvesting workers with extremely high confidence. This feedforward closed-loop strategy fundamentally ensures that heavy-duty transport robots can directly penetrate the correct high-dimensional operational corridors with minimal time latency, thereby substantially reducing the high non-productive time compensation incurred by robot clusters during blind optimization in maze-like habitats.

Finally, once agricultural machinery enters extremely narrow operational corridors, the limit control of low-level kinematics assumes responsibility for the absolute physical safety perimeter. Addressing this micro-scenario, Kulathunga et al. meticulously designed and deployed a highly modular, perception-driven closed-loop navigation foundation [150]. The front end of this control system relies on a multi-step progressive point cloud processing pipeline to achieve high-fidelity geometric extraction of the physical boundaries of complex crop rows; the back end converts these environmental features into rigorous non-holonomic vehicle dynamics constraints [151]. Empirical validation demonstrates that, by virtue of this micro-control strategy, large-scale agricultural robots can achieve high-precision trajectory servo tracking and flexible, smooth navigation—even in extreme micro-environments such as strawberry plastic greenhouses with severely constrained spaces and high collision susceptibility—at an astonishingly low average lateral deviation of only 0.08 m [152,153]. This establishes an impregnable bottom-layer execution safety defense for the entire high-density Human–Robot Collaboration (HRC) logistics relay system.

Regarding the macro-level implementation and panoramic planning of system engineering, Sánchez-Molina et al. conducted a rigorous scoping review on the full life-cycle development of smart greenhouse robots [154]. This study systematically organized and deeply deconstructed the technological evolution chain from the physical design of low-level flexible end-effectors to top-level multimodal perception and closed-loop control algorithms [155,156,157,158], fundamentally laying a solid theoretical foundation for the hardware modularization and software communication interface standardization of modern greenhouse heterogeneous logistics equipment. To achieve efficient logistics within the complex, narrow, human–robot coexistent environment of greenhouses, Cañadas-Aránega et al. designed and validated an autonomous collaborative mobile robot (CoBot) platform specifically for enclosed greenhouse environments. Built upon the ROS 2 architecture and deeply integrating high-precision habitat-based HRC logistics transport, the system provides a highly reliable paradigm for engineering implementation [159,160].

However, the successful engineering deployment of any cutting-edge agricultural cyber-physical equipment must never be isolated or constrained by the evaluation barrier of mere technical feasibility. Stepping beyond a purely robotic perspective, Moreno et al., using highly intensive European Mediterranean greenhouse clusters as a representative empirical case, performed a comprehensive feasibility analysis on the large-scale implementation of greenhouse agricultural robots, covering five high-level dimensions: technical boundaries, economic efficiency, social ethics, legal compliance, and ecological environment [29]. This multidimensional evaluation system clearly reveals that, although low-coupling operations such as autonomous wide-area spraying have achieved high Technology Readiness Levels (TRL) [161], systems must construct highly reliable human–robot ethical safety standards and ubiquitous privacy data-sharing protocols at the top level when confronting the “picking-transport” collaborative logistics segment, which is highly unstructured and involves intense human–machine interaction. Furthermore, the marginal procurement costs of intelligent equipment must be substantially reduced at the supply chain level. Only by achieving these multidimensional boundary breakthroughs can greenhouse cluster logistics scheduling systems cross the “valley of death” of industrialization and truly realize the grand blueprint of large-scale promotion and commercial profitability.

**Figure 8:**
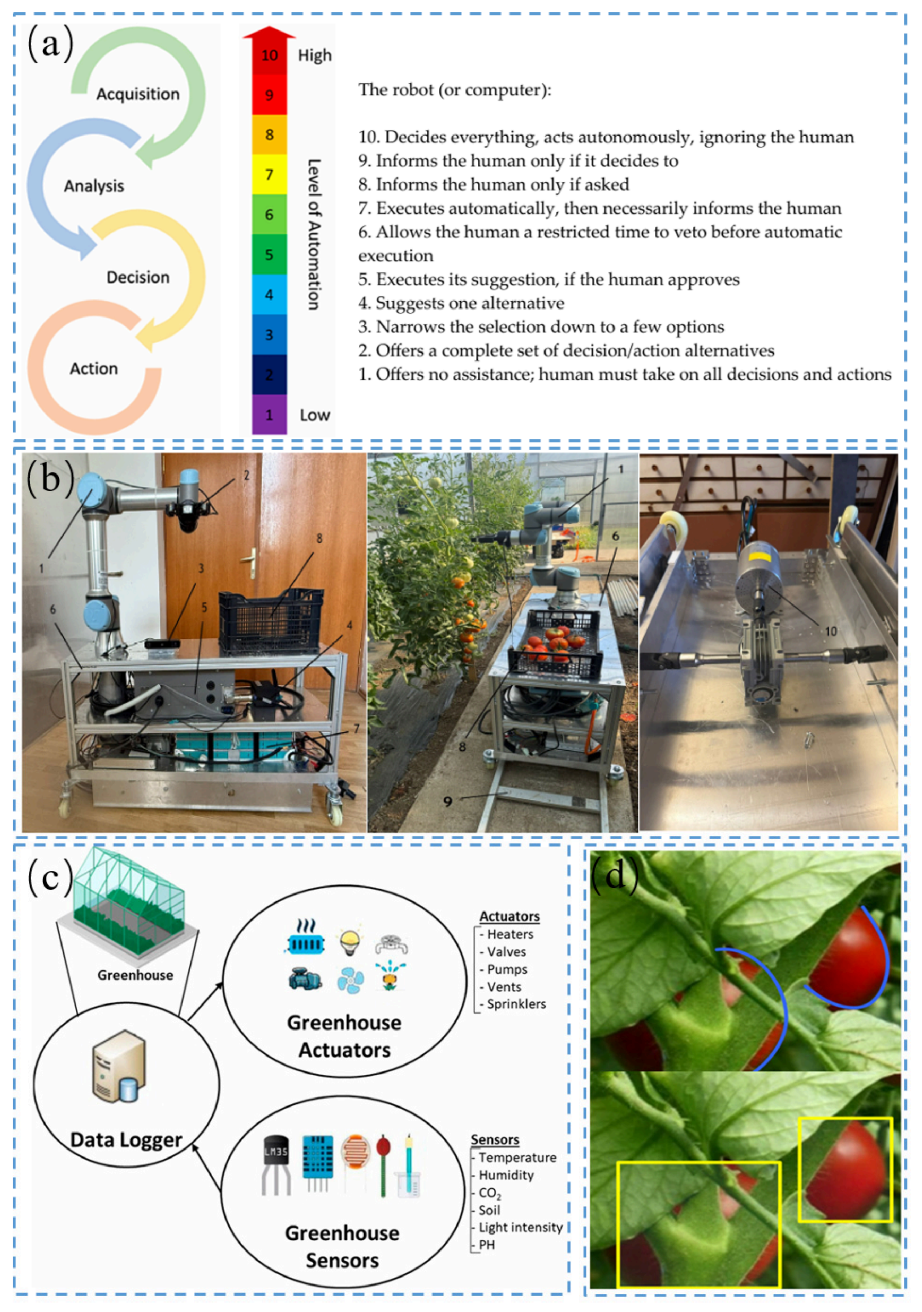
**Intelligent harvesting and Human–Robot Collaboration (HRC) paradigms in the “3D high-density” habitats of enclosed greenhouses.** (**a**) Levels of Human–Robot Interaction (HRI) from coexistence to deep collaboration and safety topologies in shared agricultural workspaces [11]. (**b**) Synergistic system of autonomous tomato harvesting robot chassis and manipulators in structured rail environments [152]. (**c**) Multi-source sensing and IoT fusion architecture in smart greenhouses supporting 3D high-density operations [39]. (**d**) Empirical results of underlying AI visual target recognition and maturity detection overcoming complex foliage occlusion and lighting interference in greenhouses [152].

Ultimately, from the highly robust, narrow-row physical traversal at the execution end, through the high-frequency interaction of human-intention that ensures absolute physical safety at the control end, to the macro-level economic efficiency evaluation and sociological ethical considerations at the system end, the aforementioned series of highly forward-looking, rigorous studies have jointly outlined and consolidated a new high-density digital logistics paradigm for modern enclosed greenhouses characterized by “front-end flexible human–robot complementarity and back-end high-dimensional vertical relay.” By integrating advanced SLAM algorithms and multi-source safety perception sensors, not only have dynamic obstacle avoidance been achieved, but challenges regarding chassis passability and driving stability for heavy-load (100 kg) agricultural machinery on uneven, undulating greenhouse surfaces have been overcome. This deep synergy, spanning the boundaries between physical entities and digital computation, unequivocally signifies that traditional greenhouse manual operation models are moving irreversibly toward a highly intelligent and ubiquitously connected Agricultural Cyber-Physical Ecology. To intuitively demonstrate these paradigms, Figure 8 illustrates the intelligent harvesting and Human–Robot Collaboration (HRC) frameworks in the ‘3D high-density’ habitats of enclosed greenhouses. Figure 8a details the levels of HRI and safety topologies in shared workspaces, while Figure 8b,c exhibit the autonomous harvesting robot chassis and the multi-source sensing IoT fusion architecture. Together with the underlying AI visual target recognition results in Figure 8d, these components unequivocally signify the technological leap towards a highly intelligent and safe human–machine ecological symbiosis.

## 6. Challenges, Future Prospects, and Conclusions

Although substantial breakthroughs have been achieved in agricultural picking-transport cluster coordination technology—spanning low-level multimodal heterogeneous perception, central high-dimensional operational research, and field validation across diverse habitats—advancing this highly coupled Agricultural Cyber-Physical System (ACPS) toward a full-scale, all-weather, and high-Technology Readiness Level (TRL) “Agriculture 4.0” commercial application still necessitates crossing a series of deep theoretical chasms and systematic engineering barriers. As the research scope expands from highly controlled laboratory environments to real-world commercial farms riddled with strong stochastic perturbations, multi-robot collaborative scheduling has completely shed its pure technical nature—previously defined by isolated mathematical optimization or low-level electromechanical servo control [162]. It has evolved into a socio-technical system challenge, deeply intertwined with rigorous multidimensional constraints such as agricultural economic efficiency assessment, social labor structural shifts, data privacy and compliance oversight, and long-term ecological succession.

Based on the aforementioned macroscopic perspective, this section elevates the viewpoint to systematically dissect the frontier systematic bottlenecks currently facing smart agricultural logistics networks on the eve of large-scale deployment (Section 5.1). The argumentative logic will rigorously transcend the framework of pure technological determinism, focusing on deconstructing physical boundaries such as limited edge computing power and communication latency, ethical and regulatory blind spots within Human–Robot Collaboration (HRC) frameworks, and future core scientific research directions such as energy-optimal and environment-friendly sustainable scheduling. On the basis of this multidimensional discussion, this section (Section 5.2) will conduct a global and highly refined distillation of the entire research thread and core theoretical evolutionary trajectory, aiming to anchor the final academic coordinates and strategic development roadmap for building a new generation of unmanned farm multi-agent collaborative scheduling networks that possess high cognitive autonomy, extreme topological flexibility, and system resilience.

### 6.1. Core Barriers and Frontier Challenges of Large-Scale Deployment

Currently, agricultural cluster scheduling systems are at a historical turning point, transitioning from the proof-of-concept phase of ideal laboratory prototypes to large-scale deployment on commercial farms. Sharma et al. provided a panoramic overview of the technological revolution in smart agriculture, explicitly stating that the deep integration of ubiquitous IoT, multi-dimensional big data, deep learning frameworks, and swarm intelligence is the core underlying logic driving modern agriculture toward the ultimate goal of “Zero Hunger” [2,163]. However, the research also issues a stern warning: this spectacular evolutionary process is encountering severe systematic tests, including data privacy breaches, cross-hierarchical cybersecurity vulnerabilities, and the lag in foundational rural digital infrastructure [164,165]. To truly achieve all-weather, high-robustness, unmanned, large-scale applications, the academic and engineering communities must still cross deep-seated systematic barriers in the following three core dimensions:

First is the deep game between comprehensive feasibility and social ethical norms. The theoretical advancement of cutting-edge technology cannot be simply equated with actual engineering feasibility. Taking high-density Mediterranean greenhouses as a typical habitat, Moreno et al. conducted a penetrating analysis of the comprehensive feasibility of agricultural collaborative robots [29]. The team sharply pointed out that the wide-area application of robot clusters must not remain solely in the self-verification of “technical feasibility” but must accept rigorous scrutiny from multiple dimensions, including economic efficiency (e.g., Return on Investment, ROI), impact on social structures (e.g., traditional labor replacement), legal compliance, and ecological environment. If cluster systems cannot form a self-consistent cost–benefit closed loop in their economic models, even the most exquisite operational scheduling algorithms will eventually become castles in the air, incapable of achieving commercial profitability [166]. Focusing on the data ownership and economic barriers, the frontier challenge is not merely establishing top-down regulations, but architecting decentralized data-sharing frameworks. Future ACPS must transition towards Agricultural Federated Learning (Ag-FL) paradigms. This would allow heterogeneous fleets across different commercial farms to collaboratively train robust perception models without ever transferring proprietary, raw yield data to centralized clouds [11,167]. Economically, to bridge the sociological divide identified by Karunathilake et al. [168], the industry must move beyond high-premium monolithic machine sales toward a modular ‘Robotics-as-a-Service’ (RaaS) scheduling ecosystem, decoupling hardware ownership from algorithmic deployment to ensure inclusivity for small-hold farmers.

Second is the prominent methodological disconnect between complex algorithmic architectures and limited edge-computing hardware. A clear mapping of current research reveals a paradoxical trend: while state-of-the-art perception models (e.g., Vision Transformers [30]) achieve unprecedented accuracy in laboratory settings, their massive parameter counts create an insurmountable bottleneck in real-world agricultural terminals [169,170]. The fundamental flaw in the recent literature is the over-prioritization of algorithmic precision at the expense of computational efficiency and passive cooling limits. Therefore, the most critical research gap moving forward is not the pursuit of deeper neural networks, but the development of fault-tolerant, heavily pruned, and highly sparse models capable of sustaining real-time closed-loop control under the strict energy constraints of autonomous fleets. Such extreme physical hardware boundaries constitute a massive engineering obstacle for cutting-edge algorithms as they cross from ideal simulation environments with infinite computing power to resource-constrained physical entities [171]. Furthermore, a glaring research gap exists in quantifying the “Sim-to-Real” performance degradation. Current literature frequently claims successful physical deployment of policies trained in simulation, yet systematically fails to provide strict quantitative analyses of performance decay across domains. For instance, studies rarely quantify how much the collision rate increases, or how the kinematic tracking error quantitatively degrades (e.g., measured in precise metric variance) when a multi-agent policy transitions from a high-fidelity physics simulator to a physical farm with unpredictable friction and actuation delays [171]. Without establishing standardized quantitative benchmarks to measure this environmental domain shift, the transition of data-driven scheduling from simulated prototypes to commercial fleets remains inherently unpredictable. Meanwhile, Talib et al., when systematically reviewing video data management models under agricultural edge computing architectures, clearly pointed out that the extremely high communication latency and network bandwidth congestion induced by the backhaul of massive, multimodal, high-dimensional perception data in cloud-edge-terminal collaboration remain the fatal engineering shortcomings restricting high-concurrency heterogeneous fleets from executing low-latency, real-time collaborative positioning and high-frequency scheduling decisions [45,172].

**Figure 9:**
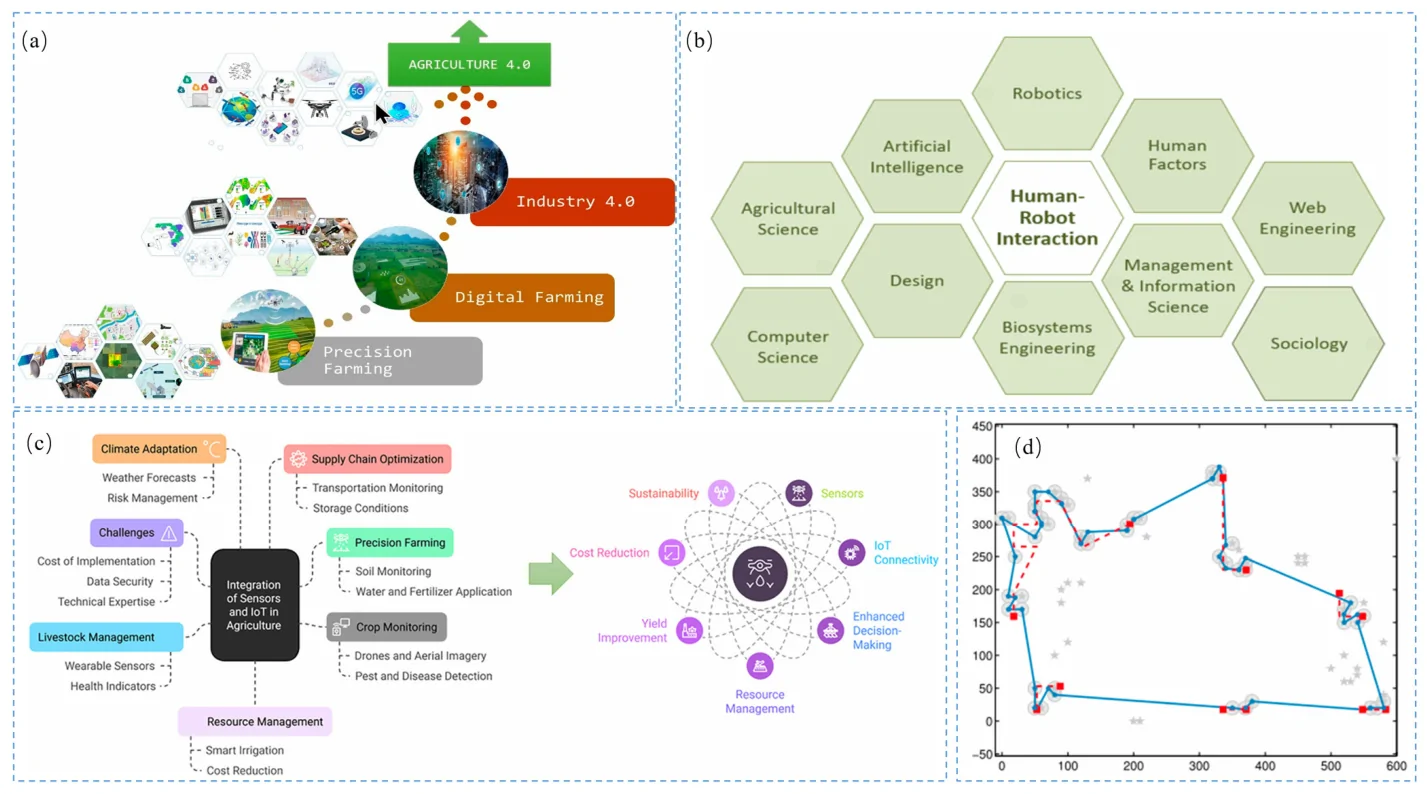
**Technological evolution and future perspectives for next-generation Agricultural Cyber-Physical Systems (ACPS).** (**a**) Agriculture 4.0 smart farming holistic ecosystem integrating AI and ubiquitous sensing [19]. (**b**) High-level “Human–Robot–Environment” co-integration evaluation and challenge framework encompassing ecological sustainability and social ethics [11]. (**c**) Future agricultural big data fusion and intelligent Decision Support System (DSS) architecture driving ultra-low latency closed-loops [37]. (**d**) Cross-domain heterogeneous (UAV-UGV) swarm collaborative topology based on multi-agent networks [107].

Finally, it is the thorough evolution toward an environment-friendly and energy-sensitive sustainable scheduling paradigm. Chuyen and Rajagopal, in their comprehensive review of global agricultural product macro-logistics networks, draw a definitive conclusion: future agricultural logistics systems must completely abandon the singular and short-sighted orientation of “time efficiency first” and shift entirely toward a multi-dimensional sustainable development framework that deeply balances economic benefits, social equity, and the long-term stability of the ecological environment [173,174]. This paradigm restructuring implies that future cluster micro-path planning and macro-task allocation can no longer pursue only the shortest travel distance or the optimal makespan in geometric space. Instead, they must incorporate the dynamic energy consumption evolution models of multi-agent heterogeneous agricultural machinery [175], the non-linear battery decay triggered by unstructured muddy resistance, and even the irreversible ecological damage caused by heavy agricultural machinery tracks to subsoil compaction as core penalty weights into the high-dimensional multi-objective fitness function for joint global optimization. Furthermore, as profoundly envisioned by Zhang et al., the next evolutionary leap in ACPS will be the transition from pure ‘spatiotemporal constraint scheduling’ to ‘Bio-Cybernetic Scheduling’. Future digital twin architectures must deeply couple real-time plant physiological stress signals (e.g., volatile organic compound emissions or sap flow fluctuations) directly into the multi-agent logistics objective functions. In this paradigm, transport capacity and collaborative harvesting sequences will no longer be dictated solely by geometric shortest paths or static yield maps, but by the real-time biological urgency of the crop itself. This deep fusion of macro-logistics with micro-biological feedback will define the ultimate performance ceiling and ecological harmony of next-generation unmanned farm collaborative networks. Looking forward, the technological evolution and future perspectives for the next-generation ACPS are comprehensively mapped out in Figure 9. As shown in Figure 9a,b, the Agriculture 4.0 ecosystem must integrate AI and ubiquitous sensing while adhering to a rigorous ‘Human–Robot–Environment’ co-integration framework that encompasses ecological sustainability and social ethics. Furthermore, Figure 9c,d highlight the future agricultural big data fusion architecture and the cross-domain UAV-UGV swarm collaborative topology, which will ultimately define the performance ceiling of next-generation unmanned farm networks.

### 6.2. Summary

In summary, under the dual macroscopic constraints of the global structural shortage of agricultural labor and the United Nations “Zero Hunger” strategic goal, collaborative “picking-transport” cluster operations in complex agricultural habitats have become the fundamental cornerstone for breaking through traditional agricultural productivity limits. This review has systematically deconstructed and provided a panoramic overview of multi-robot logistics scheduling systems in unstructured agricultural environments. In-depth research confirms that modern agricultural logistics scheduling is by no means a low-level mechanical aggregation of multi-source heterogeneous agricultural equipment in physical space, but rather a complex Agricultural Cyber-Physical System (ACPS) deeply coupled with multimodal intelligent heterogeneous perception, distributed edge computing foundations, and high-dimensional multi-agent collaborative control algorithms.

Looking at the technological evolution trajectory throughout the entire life cycle of this field, the underlying scheduling system is undergoing a profound paradigm shift. At the level of environmental data foundations, it is evolving from a heavy reliance on prior mapping—based solely on Global Navigation Satellite Systems (GNSS) and low-dimensional planar grid maps—toward distributed, robust perception integrated with LiDAR and multidimensional vision, as well as high-fidelity Digital Twin foundations deeply embedded with yield spatial predictions and three-dimensional terrain traversability cost models. At the level of core scheduling algorithms, centralized analytical operations research, which is highly dependent on static prior environments, is being deeply coupled with data-driven frameworks of Multi-Agent Reinforcement Learning (MARL), the low-level closed loops of Distributed Nonlinear Model Predictive Control (DiNMPC), and ecological assessment models incorporating soil compaction dynamics (such as SoilFlex). This is aggressively driving the system’s decision-making optimization to shift from “passive rule-based response, exclusively pursuing spatiotemporal efficiency” toward a macro-multidimensional sublimation of “high-dimensional feedforward prediction, deeply balancing ecological sustainability.” At the level of scene implementation and engineering practice, from high-speed, non-stop dynamic escort and relay in continuous flows of vast fields, to flexible shuttle obstacle avoidance in extremely narrow row spacings of dense orchards, and finally to high-level Human–Robot Collaboration (HRC) ensuring absolute physical safety in the three-dimensional high-density spaces of enclosed greenhouses, multi-robot collaborative scheduling networks have initially demonstrated powerful engineering generalization and adaptive capabilities in crossing the boundaries of extremely unstructured physical habitats.

However, moving toward the ultimate vision of fully cognitive autonomous and ubiquitously connected next-generation unmanned farms (Agriculture 4.0) remains a massive systematic engineering project fraught with significant engineering urgency and interdisciplinary challenges [176]. As profoundly revealed by the analysis in this paper, fully crossing the edge computing and communication bandwidth gap from “Sim-to-Real,” accelerating the establishment of ethical and regulatory frameworks for multi-party data ownership and sharing, and rigorously verifying the comprehensive cost–benefit closed loop of expensive agricultural cyber-physical equipment are “deep-water zone” challenges that the entire industry must face in the next stage of breaking through large-scale deployment. It is foreseeable that with the continuous iterative evolution of underlying generalized AI algorithm models and the increasing perfection of wide-area smart agricultural IoT infrastructure [177], a panoramic agricultural logistics scheduling network—possessing high cognitive autonomy, extreme network topological resilience, and a highly harmonious human–machine ecological symbiosis—will inevitably reshape the underlying spatiotemporal operational logic of global agricultural digital production in the near future.

In terms of technological prospects, three categories of methods demonstrate the most transformative application potential for next-generation agricultural harvest–transport logistics systems. First, the deeply coupled architecture of multi-modal semantic perception and digital twins, represented by LiDAR-vision fusion and traversability cost modeling: It converts raw geometric point clouds of unstructured farmland into schedulable semantic twin models embedded with physical constraints, serving as the core data foundation for all upper-level scheduling decisions, and will remain a long-term research focus at the perception layer. Second, the hybrid scheduling paradigm that integrates centralized operations research optimization and decentralized multi-agent reinforcement learning: This framework balances the global optimality of macro resource allocation and the dynamic adaptability of micro real-time response, and is currently the most feasible technical route to promote the large-scale deployment of Agricultural Cyber-Physical Systems from laboratories to commercial farms. Third, the ecologically sustainable scheduling mechanism represented by soil compaction minimization and energy consumption optimization: It breaks through the single objective of traditional scheduling that only pursues time efficiency, and incorporates cultivated land protection and ecological costs into the scheduling objective function. In line with the global trend of conservation tillage and sustainable agriculture, this dimension will become an indispensable core component of next-generation agricultural logistics scheduling systems.

## Figures and Tables

**Table 1:** Representative Perception and Localization Technologies for Agricultural Robots in Unstructured Environments.

Challenge Dimension	Specific Bottlenecks	Potential Breakthrough Directions	References
Economic and socio-ethical barriers	Unclosed cost–benefit loop; high equipment premium; lack of HRC safety regulations; unclear data ownership	Standardize HRC ethical guidelines; reduce hardware costs via modular design; establish multi-stakeholder data sharing mechanisms	[29]
Edge computing and sim-to-real gap	Limited on-board computing power; high latency of massive perception data; poor cross-domain generalization of simulation-trained models	Lightweight model pruning and quantization; edge-cloud collaborative inference; domain adaptation and sim-to-real transfer learning	[30]
Ecological sustainability	Single-minded pursuit of time efficiency; soil compaction from heavy machinery; non-linear energy consumption on uneven terrain	Integrate soil compaction and energy consumption into multi-objective scheduling; build energy-aware digital twin models	[10]
Digital twin and full-chain management	Insufficient digital twin modeling accuracy; difficulties in multi-source data fusion; weak closed-loop decision-making capability	Construct high-fidelity digital twin bodies for agricultural machinery; fuse multi-modal sensing data for virtual-real interactive optimization	[13]
Human–robot interaction efficiency	Lack of natural HRI modalities; high operation threshold for field workers; unreasonable human–robot task allocation	Develop multi-modal natural HRI interfaces; optimize human–robot task allocation mechanisms; improve system usability	[11]

**Table 2:** Comparison of Path Planning and Obstacle Avoidance Algorithms for Agricultural Mobile Robots.

Algorithmic Paradigm	Core Methods	Core Advantages	Main Limitations	Typical Application	References
Sampling-based path search	Continuous bidirectional Quick-RRT with non-holonomic kinematic constraints	Fast solution speed and high path smoothness in narrow orchard passages	Prone to local optimum in highly dense obstacle scenes	Narrow-row orchard shuttling and multi-robot deadlock recovery	[62]
Hybrid dynamic window approach	TD3 reinforcement learning + DWA hybrid framework	Strong adaptability to unknown dynamic environments with feedforward predictive obstacle avoidance	Poor interpretability and noticeable sim-to-real gap	Dynamic obstacle avoidance and multi-agent formation keeping	[18]
Headland turning trajectory planning	Multi-constraint safe trajectory planning with obstacle awareness for cluttered orchards	Satisfies non-holonomic constraints and improves safety and efficiency of headland maneuvers	Insufficient adaptability in extremely narrow headland scenarios	Autonomous agricultural vehicle headland turning operations	[63]
Ecological coverage path planning	Soil2Cover framework with embedded SoilFlex compaction model	Reduces headland soil compaction by up to 30% by integrating ecological cost into objectives	Increases solution space complexity and requires soil mechanical parameters	Open-field coverage path planning and heavy machinery traffic control	[10]
End-to-end reinforcement learning	Deep reinforcement learning with farm-tailored reward function	Requires no prior environmental map and autonomously learns optimal policies	High training cost and weak decision interpretability	Autonomous exploration and obstacle avoidance in unknown farmland	[19]
Interval type-2 fuzzy control	IT-2 fuzzy logic + behavior-based navigation architecture	High robustness to sensor noise and compatibility with 4WD4WS holonomic robots	Parameter tuning relies heavily on expert experience	High-precision path tracking in unstructured fields	[64]

**Table 3:** Cooperative Scheduling and Motion Control Methods for Agricultural Machinery Fleets.

Algorithm Category	Representative Methods	Core Optimization Objectives	Applicable Scenario	References
Meta-heuristic global scheduling	NSGA-III + improved ant colony optimization	Multi-objective minimization of operation makespan, travel distance and labor cost	Open-field multi-machine cooperative task allocation	[8]
Harvester-transport cooperative scheduling	Improved mayfly optimization algorithm (IMPA)	Minimization of total operation time and empty driving distance	Combine harvester and grain cart cooperative harvesting	[93]
Distributed model predictive control	DiNMPC distributed nonlinear model predictive control	High-precision trajectory tracking and articulated dynamics handling	Tractor-trailer path following and side-by-side unloading-on-the-go	[94]
Leader–follower coordinated control	Neuro-adaptive PID + leader–follower architecture	Velocity and pose synchronization for stable on-the-go unloading	Parallel operation of combine harvesters and transport carts	[95]
Decentralized fleet routing	Agriculture Fleet Vehicle Routing Problem (AF-VRP)	Cross-farm resource sharing, fairness and scalability	Wide-area multi-farm dynamic task allocation and equipment sharing	[12]
Multi-UAV formation planning	Multi-agent reinforcement learning (SAC) with decentralized decision making	Formation maintenance stability and dynamic environment adaptability	Farmland multi-UAV inspection and crop protection	[21]

## Data Availability

No new data were created or analyzed in this study. Data sharing is not applicable to this article.
